# Enhanced chromatin accessibility of the dosage compensated *Drosophila* male X-chromosome requires the CLAMP zinc finger protein

**DOI:** 10.1371/journal.pone.0186855

**Published:** 2017-10-27

**Authors:** Jennifer Urban, Guray Kuzu, Sarah Bowman, Benjamin Scruggs, Telmo Henriques, Robert Kingston, Karen Adelman, Michael Tolstorukov, Erica Larschan

**Affiliations:** 1 Department of Molecular Biology, Cell Biology, and Biochemistry, Brown University, Providence, Rhode Island, United States of America; 2 Department of Molecular Biology, Massachusetts General Hospital, Boston, Massachusetts, United States of America; 3 Epigenetics and Stem Cell Biology Laboratory, National Institute of Environmental Health Sciences, NIH, North Carolina, United States of America; 4 Department of Genetics, Harvard Medical School, Boston, Massachusetts, United States of America; Max Planck Institute of Immunobiology and Epigenetics, GERMANY

## Abstract

The essential process of dosage compensation is required to equalize gene expression of X-chromosome genes between males (XY) and females (XX). In *Drosophila*, the conserved Male-specific lethal (MSL) histone acetyltransferase complex mediates dosage compensation by increasing transcript levels from genes on the single male X-chromosome approximately two-fold. Consistent with its increased levels of transcription, the male X-chromosome has enhanced chromatin accessibility, distinguishing it from the autosomes. Here, we demonstrate that the non-sex-specific CLAMP (Chromatin-linked adaptor for MSL proteins) zinc finger protein that recognizes GA-rich sequences genome-wide promotes the specialized chromatin environment on the male X-chromosome and can act over long genomic distances (~14 kb). Although MSL complex is required for increasing transcript levels of X-linked genes, it is not required for enhancing global male X-chromosome chromatin accessibility, and instead works cooperatively with CLAMP to facilitate an accessible chromatin configuration at its sites of highest occupancy. Furthermore, CLAMP regulates chromatin structure at strong MSL complex binding sites through promoting recruitment of the Nucleosome Remodeling Factor (NURF) complex. In contrast to the X-chromosome, CLAMP regulates chromatin and gene expression on autosomes through a distinct mechanism that does not involve NURF recruitment. Overall, our results support a model where synergy between a non-sex-specific transcription factor (CLAMP) and a sex-specific cofactor (MSL) creates a specialized chromatin domain on the male X-chromosome.

## Introduction

Dosage compensation is an ancient mechanism that functions to regulate transcription of X-chromosome genes to equalize transcript levels between XY males and XX females. There is mounting evidence that one conserved mechanism to equalize gene dosage is upregulation of the X-chromosome [1]. In fact, in *D*. *melanogaster* and across *Diptera*, dosage compensation occurs by increasing transcript levels of thousands of genes along the length of the single male X-chromosome two-fold to equalize transcript levels with XX females [2–4]. This process of increasing X-chromosome transcript levels is mediated by the Male-specific lethal (MSL) histone acetyltransferase complex [3].

It was long thought that the MSL complex promotes an open chromatin environment on the X-chromosome through the H4K16 acetyltransferase activity of its Males absent on the First (MOF) component in order to facilitate increased transcription [5]. The acetylated H4K16 histone modification directly interferes with chromatin structure by disrupting interactions between nucleosomes, causing chromatin to unfold [6]. However, a recent study using Micrococcal Nuclease sequencing (MNase-seq) found that the MSL complex has only a minor role in opening chromatin, which occurs specifically at its sites of highest occupancy called Chromatin Entry Sites (CES) [7]. This study additionally found that the three-dimensional chromosome organization of the X-chromosome is similar in males and females and forms independently of MSL complex. Furthermore, induction of the MSL complex in females leads to recognition of the same X-chromosome binding sites that are occupied in males [8]. Together, these observations led us to hypothesize that a non-sex-specific factor functions upstream of MSL complex to establish the enhanced chromatin accessibility that allows MSL complex to distinguish the male X-chromosome from autosomes.

We previously discovered that a non-sex-specific transcription factor, which we named Chromatin-linked adapter for MSL Proteins (CLAMP), is required for MSL complex recruitment to the male X-chromosome [9,10]. CLAMP directly binds to GA-rich *cis*-elements located within CES, both in the presence and absence of MSL complex [9,11]. On the X-chromosome, evolutionary expansion of GA-repeats increased the number and affinity of CLAMP binding sites [11]. These expanded GA-rich *cis*-elements are more clustered within CES than anywhere else in the genome [11]. Together, the increased number and density of GA-rich *cis*-elements elevate CLAMP occupancy on the X-chromosome compared with autosomes [9,11]. MSL complex further increases CLAMP occupancy at approximately 2/3 of CES, suggesting that CLAMP and MSL complex bind synergistically at a subset of their binding sites [9].

In both males and females, CLAMP binds more frequently within gene bodies on the X-chromosome than autosomes [11] because CES are located toward the 3’ end of active X-linked gene bodies [12]. In contrast, autosomes have lower CLAMP occupancy levels and primarily single binding sites that are enriched within promoters [9]. Furthermore, polytene chromosomes from *clamp* null mutant larvae exhibit disrupted chromatin structure [10], suggesting a possible role for CLAMP in establishing patterns of chromatin accessibility. Based on these observations, we hypothesized that the clusters of CLAMP binding sites that exist more frequently over gene bodies on the X-chromosome than autosomes make CLAMP a strong candidate for promoting the enhanced chromatin accessibility that targets MSL complex to the X-chromosome.

Here, we use genomic approaches to demonstrate that CLAMP can act over substantial genomic distances (~14 kb) to promote the formation of a broad domain of enhanced chromatin accessibility on the male X-chromosome. In contrast, MSL complex functions to increase chromatin accessibility specifically at CES peak centers, which are its sites of highest occupancy. Interestingly, CLAMP modulates chromatin accessibility over significant distances beyond the center of CES independent of MSL complex. At a subset of CES where CLAMP and MSL complex promote each other’s occupancy [9], CLAMP increases recruitment of the Nucleosome Remodeling Factor (NURF) complex and reduces histone occupancy. In contrast, we demonstrate that CLAMP influences the position of chromatin accessibility near transcription start sites through a mechanism that does not involve the NURF complex or modulating histone occupancy. Overall, we identify two separable context-specific roles for a single transcription factor: 1) CLAMP functions via MSL-dependent and independent mechanisms to promote the formation of a specialized chromatin domain on the male X-chromosome; 2) CLAMP regulates chromatin accessibility at promoters and transcription termination sites genome-wide using a different mechanism that does not involve altering NURF or histone occupancy.

## Results

### CLAMP promotes globally enhanced chromatin accessibility of the male X-chromosome

**Fig 1:**
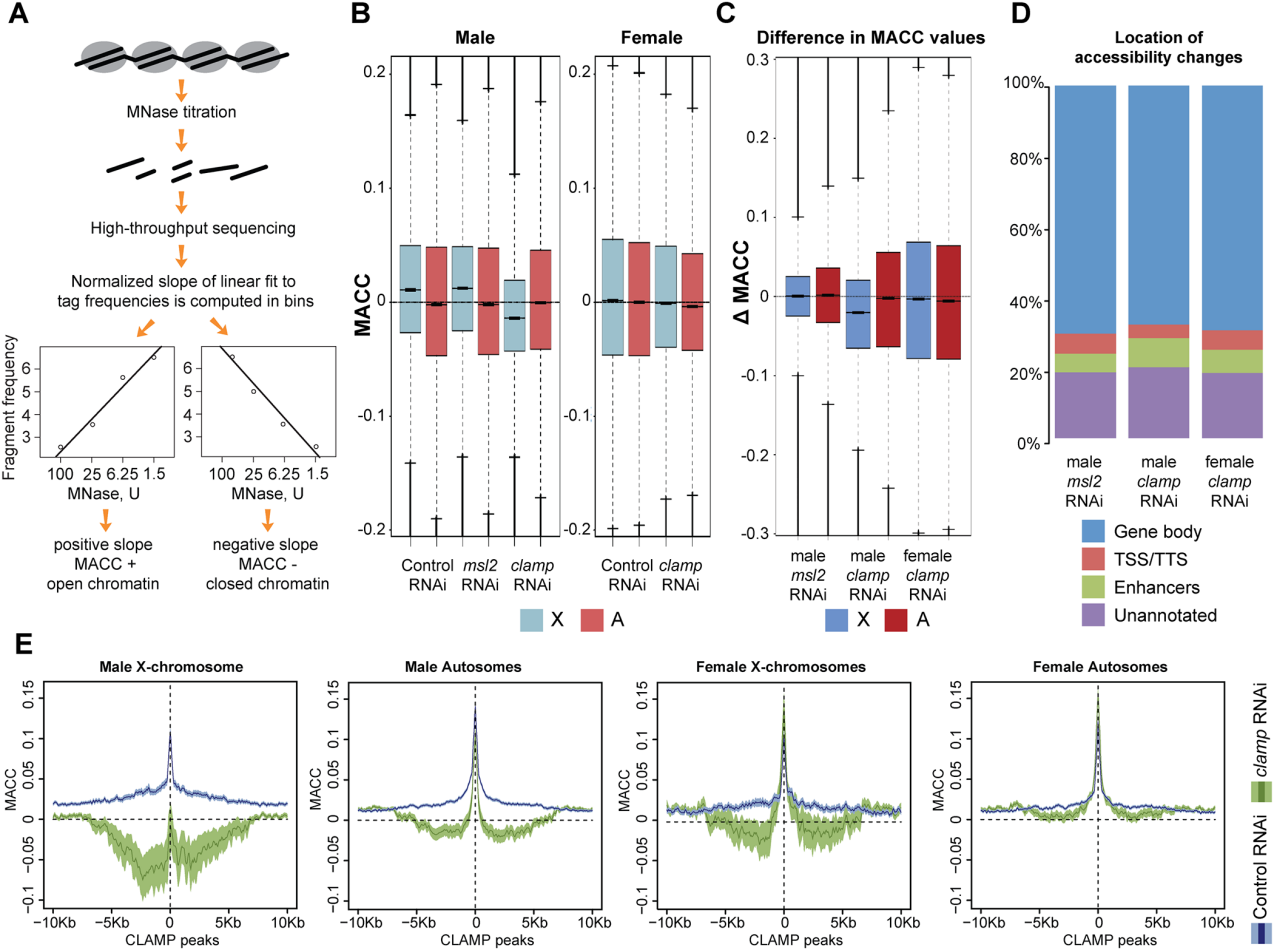
CLAMP establishes the global open chromatin environment on the male X-chromosome. A) Chromatin is separated into four samples which are digested under differing concentrations of MNase. The digested samples are prepared for high-throughput sequencing and the number of reads obtained within a region of the genome is plotted for all MNase concentrations from highest to lowest. A linear regression line is fit to the data points, and the slope of the line derives the MNase accessibility (MACC) score. B) The overall distribution of accessibility scores measured by the MACC value is shown for the X-chromosome (blue) and autosomes (red) of Control (*gfp*), *clamp*, and *msl2* RNAi treated male (S2) and female (Kc) cells. The male X-chromosome has overall higher MACC values than the autosomes. This accessibility is reduced after *clamp* RNAi. In females, MACC values for the X-chromosomes and autosomes are similar and are reduced slightly following *clamp* RNAi treatment. For all box and whisker plots, the 95% confidence interval is shown with a notch around the median line. C) For both the X-chromosomes and autosomes, the difference in MACC value (Δ MACC) between control and RNAi treatment for an individual 100bp bin was calculated to account for differences in chromosome number. In males, the change in MACC scores indicates a reduction in X-chromosome accessibility following *clamp* RNAi but not *msl2* RNAi. D) The location of significant changes in accessibility (p-value < 0.01) was classified as either within a gene body (blue), at TSS or TTS (red), at enhancers (green), or in unannotated regions (purple). Details for region definitions are in the Methods. For all RNAi treatments, the largest proportion of accessibility changes is located within gene bodies. E) Average MACC values were plotted over a 20kb window centered on a CLAMP ChIP-seq peak [9] for both control RNAi (blue) and *clamp* RNAi (green) conditions. The lighter shading surrounding the darker average line represents the 95% confidence interval. On both male and female X-chromosomes there is a strong reduction in accessibility that extends approximates +/- 7kb from the peak center. A similar, but less pronounced effect is also seen on autosomes in males and females.

To examine whether CLAMP regulates chromatin accessibility, we identified nuclease-accessible regions genome-wide using a recently developed assay that is based on chromatin digestion in a MNase titration series followed by high-throughput sequencing [13] (Fig 1A). Using a range of concentrations of MNase enzyme provides richer information as compared to digestion with a single concentration [13–18]. After sequencing of digested fragments, the number of reads for any specific region of the genome is computed for each MNase titration point (four points arranged from highest to lowest concentration) and a linear regression line is fit to the data. The normalized slope of the line provides a metric called the MNase accessibility (MACC) score that represents how readily an individual region of the genome is digested with MNase. Regions of open chromatin are easily accessible to MNase at low enzyme concentrations (which results in high read counts) and become over-digested at higher MNase concentrations (low read counts). This produces a positive slope of the regression line, resulting in positive MACC scores (Fig 1A). In contrast, regions that are less accessible require increased amounts of MNase enzyme to produce high read counts for nucleosome-size fragments, resulting in negative MACC scores (Fig 1A). In this way, the MACC assay quantitatively profiles both open and closed regions of chromatin simultaneously, an advantage compared with other currently available methods for profiling chromatin accessibility [19].

To test our hypothesis that CLAMP promotes global opening of the chromatin on the male X-chromosome, we performed MNase-seq titrations in *Drosophila* male (S2) and female (Kc) tissue culture cells. S2 and Kc tissue culture cells are often compared to study *Drosophila* dosage compensation [20,21] because they provide a more uniform cell population than whole animals or tissues. Moreover, the MSL complex binding sites in cell culture are almost identical to those in the whole organism [21]. We reduced protein levels of CLAMP or the core MSL complex component, MSL2, in S2 cells using an RNAi strategy reported to be highly efficient in several previous studies [9,20,22]. Furthermore, we performed *clamp* RNAi but not *msl2* RNAi in Kc cells because MSL complex is not present in these female cells. As a control, we used a *gfp* RNAi treatment that activates the RNAi pathway but does not target any gene in the *Drosophila* genome [9].

We first tested *clamp* RNAi efficiency using immuno-blotting and determined that no detectable CLAMP protein remains after *clamp* RNAi as we have previously reported (S1A Fig) [9]. To test efficiency of *msl2* RNAi, we used qRT-PCR because MSL2 is an unstable protein (S1B Fig). In addition, we quantified residual MSL complex function by measuring abundance of *roX2*, which is transcriptionally activated and stabilized by MSL complex (S1C Fig). We determined that in addition to reduced *msl2* transcript, little *roX2* remained, consistent with strong MSL complex depletion (S1B and S1C Fig) [23].

After validating RNAi efficiency, we proceeded to investigate how chromatin accessibility changes in response to depletion of CLAMP or MSL complex. We first examined the overall distribution of MNase accessibility (MACC) scores within an experiment to compare cell types and conditions on a global level (S2A Fig). Additionally, we plotted MACC scores for biological replicates separately and determined that our replicates were in agreement with each other (S2B Fig). We next separated MACC values for the X-chromosome and autosomes in male (S2) and female (Kc) cells to investigate differences in accessibility between chromosomes (Fig 1B, S2C and S2D Fig). The 95% confidence intervals are shown with notches around the median lines in all box plots and therefore the lack of overlap between notches or reference lines indicates that differences between samples are statistically significant. The significance levels of MACC score differences were further evaluated by a Mann-Whitney test (see S1 Table for p-values). On average, we observed higher accessibility scores for the male X-chromosome compared to autosomes in our control RNAi condition, consistent with the hyper-transcription of this chromosome (Fig 1B and S2C Fig, control RNAi).

Relative to the control RNAi, *msl2* RNAi had no effect on the average chromatin accessibility scores of the X-chromosome or autosomes in agreement with a recent study [7] (Fig 1B and S2C Fig, *msl2* RNAi). In contrast, *clamp* RNAi reduced accessibility scores of the male X-chromosome to levels below that of autosomes (Fig 1B and S2C Fig, *clamp* RNAi). In females, *clamp* RNAi had no average effect on autosomes or the X-chromosomes relative to control RNAi (Fig 1B and S2D Fig). We further performed a direct pair-wise comparison of the accessibility scores within 100-bp bins between the control and *clamp* or *msl2* depleted samples. This approach showed that *clamp* RNAi treatment resulted in significantly more genomic locations (bins) with decreased accessibility than an increased accessibility on the male X-chromosome compared with the autosomes (Fig 1C and S2E Fig). Therefore, CLAMP and not MSL complex promotes the globally enhanced accessibility of chromatin on the male X-chromosome compared with autosomes.

Next, we performed additional analyses that further validated our observation that *clamp* RNAi specifically decreases chromatin accessibility of the male X-chromosome. First, to rule out a possibility of global differences in MACC values between samples, we determined that the average MACC profiles around a random set of genomic locations are similar for different RNAi conditions (S5B Fig). Second, we determined that *clamp* RNAi treatment decreases accessibility of the male X-chromosome when MACC values were median-shifted to zero for each sample independently (S2F Fig). Third, to check if the presence of a single male X-chromosome in male cells compared to two female X-chromosomes and two copies of each autosome can be a source of artifacts in the MACC analysis, we recomputed MACC values counting each X-linked read twice to compensate for the single X-chromosome in males (S2G Fig).

Even though global shifts in chromatin accessibility were only observed on the male X-chromosome after *clamp* RNAi (Fig 1B), many changes in chromatin accessibility occur throughout the genome after *clamp* and *msl2* RNAi treatments. To further characterize these changes in accessibility, we categorized genomic loci with significant (p-value < 0.01) changes in accessibility based on their location: within gene bodies, at transcription start or termination sites (TSS/TTS), at enhancers, or in unannotated regions (see Methods for details on region definitions). We found that the majority of accessibility changes for both *clamp* and *msl2* RNAi treatments occur within gene bodies (>65% for both treatments), consistent with the most frequent genomic location of CLAMP and MSL complex binding sites (Fig 1D). To account for differences in size of each genomic feature investigated, we also normalized counts of loci with accessibility changes by the percentage of the genome covered by each feature. Following *clamp* RNAi treatment in females, there is a roughly equal distribution of accessibility changes amongst the different genomic features (S3A Fig). In males, *clamp* RNAi results in an over-representation of changes at enhancers, indicating that CLAMP may have a role in regulating chromatin organization at these sites (S3A Fig). We conclude that CLAMP alters chromatin accessibility at a diverse set of genomic loci.

### CLAMP and MSL complex both mediate chromatin accessibility changes at CES

Diverse locus-specific changes are often masked when investigating global effects in a genome- or chromosome-wide manner. Therefore, we next investigated how accessibility changes locally at specific sites in response to *clamp* RNAi. We first generated average profiles to examine MACC scores around previously defined CLAMP ChIP-seq peaks [9] (Fig 1E, the average MACC value is shown with a thick line and 95% confidence intervals are shown as lighter shading around it). Targeting *clamp* by RNAi has a long distance effect on chromatin accessibility that extends approximately +/-7kb from the peak center. This influence on chromatin accessibility over long distances occurs independent of MSL complex because we observe similar effects on male autosomes and in females. Additionally, CLAMP functions more strongly at regions surrounding CLAMP peaks on the X-chromosome compared with autosomes, which is consistent with the non-sex-specific enrichment of CLAMP on this chromosome [9]. Interestingly, in females, the long-distance effect does not mirror the effect directly at the CLAMP peak. While CLAMP enhances chromatin accessibility beyond peak centers, it modestly represses chromatin accessibility at its peak centers, suggesting that CLAMP differentially regulates chromatin depending on the distance from the peak center. It is possible that CLAMP acts with different cofactors to modulate its function at peak centers compared with its longer range effects on chromatin accessibility [24,25].

To test whether changes in accessibility correlate with CLAMP occupancy levels, we further integrated our previously generated CLAMP ChIP-seq data [9] with the MACC data. We found that in general, regions with the greatest CLAMP occupancy tended to have higher accessibility values (S3B Fig). This property is not unique to CLAMP because the correlation between factor occupancy level and degree of chromatin accessibility has been previously demonstrated in *Drosophila* embryos [26]. However, we observed this trend in both males and females and found it was not specific to the X-chromosome (S3B Fig). Overall, the correlation between increased CLAMP occupancy and increased MACC scores on all chromosomes in both males and females suggests that this relationship is present genome-wide.

**Fig 2:**
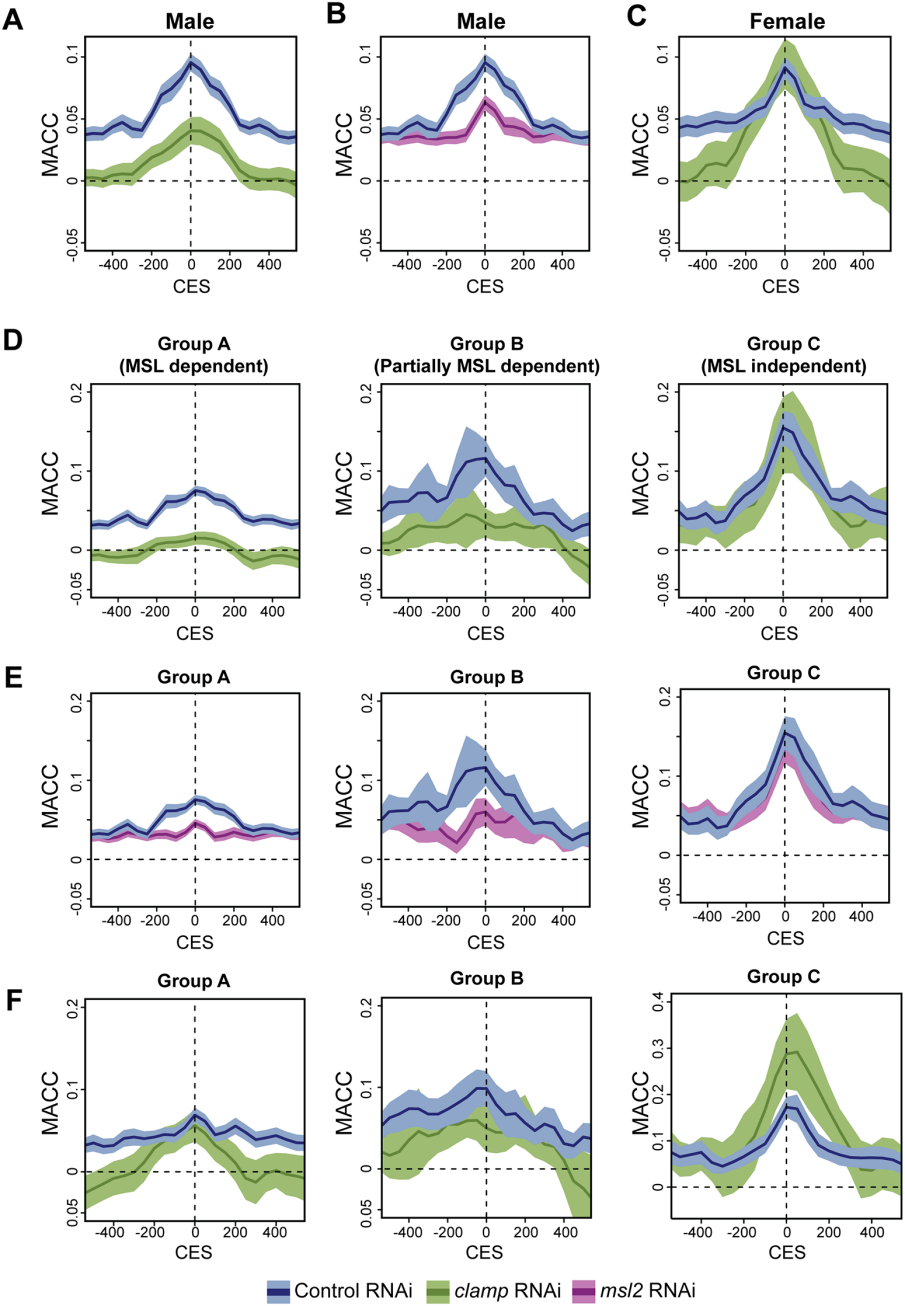
CLAMP and MSL complex promote accessibility at CES. A) MACC values at Chromatin Entry Sites (CES) in male (S2) cells are greatest at the peak center (control RNAi, blue). RNAi treatment of *clamp* reduces accessibility at the CES peak and +/- 500 bp beyond the peak (green). For all panels, the lighter color indicates 95% confidence intervals, while the darker line represents the average MACC value. B.) A reduction in MSL complex following *msl2* RNAi only reduces accessibility directly at the CES peak (purple). C) The average MACC scores around CES in female Kc cells indicates a reduction in MACC values distal to the CES center, extending +/- 500bp beyond the peak. D) MACC scores at the three subgroups of CES indicate *clamp* RNAi (green) results in a decrease in accessibility in CES Groups A and B, but not in Group C. E) RNAi targeting *msl2* (purple) results in a loss of accessibility at Group A CES only at the CES peak. There is a small reduction in accessibility in Group B sites and no effect of *msl2* RNAi treatment at Group C sites. F) MACC scores from Kc cells are plotted for the three subgroups of CES. In females, *clamp* RNAi (green) results in a decrease in MACC values around CES in Group A, and to a lesser extent Group B. CES in Group C exhibit an increase in MACC values following *clamp* RNAi.

Due to their high levels of enrichment for CLAMP and MSL complex, we examined chromatin accessibility changes at CES in response to *clamp* and *msl2* RNAi treatments. Using MACC scores from 264 CES [9], we plotted the average MACC value +/- 500bp around the CES center (dark lines in all plots in Fig 2). We also calculated the 95% confidence intervals for all profiles, which are represented by the lighter shading surrounding the central average lines. We compared the effects of CLAMP on chromatin accessibility at CES to a randomized control data set (S3C Fig) to determine the specificity of the observed effects. In male cells treated with control RNAi, chromatin was most accessible at CES centers compared to neighboring regions and this accessibility was reduced after either *clamp* or *msl2* RNAi (Fig 2A and 2B, see S1 Table for p-values). There was a focused reduction in accessibility directly at CES centers following *msl2* RNAi treatment, suggesting a specific role for MSL complex in promoting chromatin accessibility at CES centers (Fig 2B). In contrast, we observed decreases in accessibility after *clamp* RNAi in male cells at and beyond CES centers, consistent with the ability of CLAMP to promote chromatin accessibility changes over long distances from its site of occupancy (Fig 1E).

In female cells, *clamp* RNAi reduced chromatin accessibility outside of CES but not at CES centers, suggesting an MSL-independent function for CLAMP in promoting chromatin accessibility beyond the CES center (Fig 2A and 2C). The effect of *clamp* RNAi at CES in female cells is similar to what we observed on average for all CLAMP peaks (Fig 1E). Our data suggest that while MSL complex functions to promote chromatin accessibility at CES centers, CLAMP functions independently of MSL complex to promote accessibility outside of CES peak centers.

Next, we investigated the effect of *clamp* and *msl2* RNAi at previously established subclasses of CES that differ with respect to the inter-dependent binding relationship between CLAMP and MSL complex [9]. CES were previously categorized into three subgroups based on the dependence of CLAMP occupancy on MSL complex [9]: 1) At Group A CES (176 sites), CLAMP occupancy is fully dependent on the presence of MSL complex; 2) At Group B CES (43 sites), CLAMP occupancy is partially dependent on MSL complex; 3) At Group C CES (45 sites), CLAMP occupancy is independent of MSL complex. To determine differences in accessibility between the three subclasses of CES, we examined how either *clamp* or *msl2* RNAi affects average CES chromatin accessibility for each group.

We found that both *clamp* and *msl2* RNAi reduced chromatin accessibility at Group A CES most strongly followed by Group B CES (Fig 2D and 2E, Groups A and B, see S1 Table for p-values). While *msl2* RNAi reduced accessibility directly at the CES center, *clamp* RNAi reduced accessibility extending at least 500-bp beyond the center for both Group A and Group B CES. This effect is consistent with our earlier observation that CLAMP promotes chromatin accessibility over long distances (Fig 1E). Furthermore, the enhanced ability of CLAMP and MSL complex to promote chromatin accessibility at Group A and B CES is consistent with our previous hypothesis that establishing chromatin accessibility at CES requires the coordinated recruitment of CLAMP and MSL complex [9]. In contrast to Group A and B CES, chromatin accessibility at Group C CES was largely unaffected by either *clamp* or *msl2* RNAi (Fig 2D and 2E, Group C). Therefore, in the absence of MSL complex, CLAMP does not affect chromatin accessibility at Group C sites, possibly due to redundancy with another similar factor. We conclude that while both CLAMP and MSL complex promote chromatin accessibility at the majority of CES in male cells, there is a small subset of CES (Group C) that regulate their accessibility via CLAMP- and MSL-independent mechanisms.

In female cells, *clamp* RNAi affected chromatin accessibility at CES relative to control RNAi treatment, but the changes in accessibility differed from those observed in male cells. At the CES that largely require MSL complex for enhanced CLAMP occupancy (Group A CES), *clamp* RNAi reduced chromatin accessibility outside of the CES peak but not at the peak (Fig 2F, Group A). This suggests that in males MSL complex and CLAMP function together at Group A CES to promote chromatin accessibility, but in females CLAMP functions in an MSL-independent manner outside of CES centers. In contrast to other classes of CES, *clamp* RNAi enhanced chromatin accessibility at Group C CES in females (Fig 2F, Group C), similar to the average effect observed at all CLAMP peaks in females (Fig 1E). It is likely that CLAMP interacts with different co-factors in males and females, such as MSL complex, to differentially regulate chromatin accessibility at the same locus. Consistent with this hypothesis, we previously reported that CLAMP can have opposing roles in gene regulation at the same locus in males versus females [10]. Overall, we conclude that in males, MSL complex regulates chromatin accessibility precisely at CES centers, whereas CLAMP has a broader role in regulating accessibility beyond the centers of CES that is independent of MSL complex.

Recently, a new subgroup of pioneer CES called PionX sites was identified at which MSL complex is hypothesized to directly contact DNA [27]. Out of 43 PionX sites located on the X-chromosome, 23 are Group A CES. We found that at PionX sites, there was a modest decrease in accessibility that was stronger after *clamp* RNAi than *msl2* RNAi (S3B Fig, see S1 Table for p-value). These results suggest that CLAMP promotes an open chromatin environment at PionX sites that may allow MSL complex to directly contact DNA.

### CLAMP promotes changes in chromatin accessibility over X-linked gene bodies

To define how CLAMP and MSL complex contribute to chromatin accessibility within and surrounding genes, we rank-ordered all annotated genes by CLAMP ChIP-seq occupancy levels and generated heat maps of MACC scores across gene bodies (S4A Fig). We found that regions with higher CLAMP occupancy exhibited enhanced chromatin accessibility at TSS and TTS under control RNAi conditions in both males and females (S4A Fig, Control).

**Fig 3:**
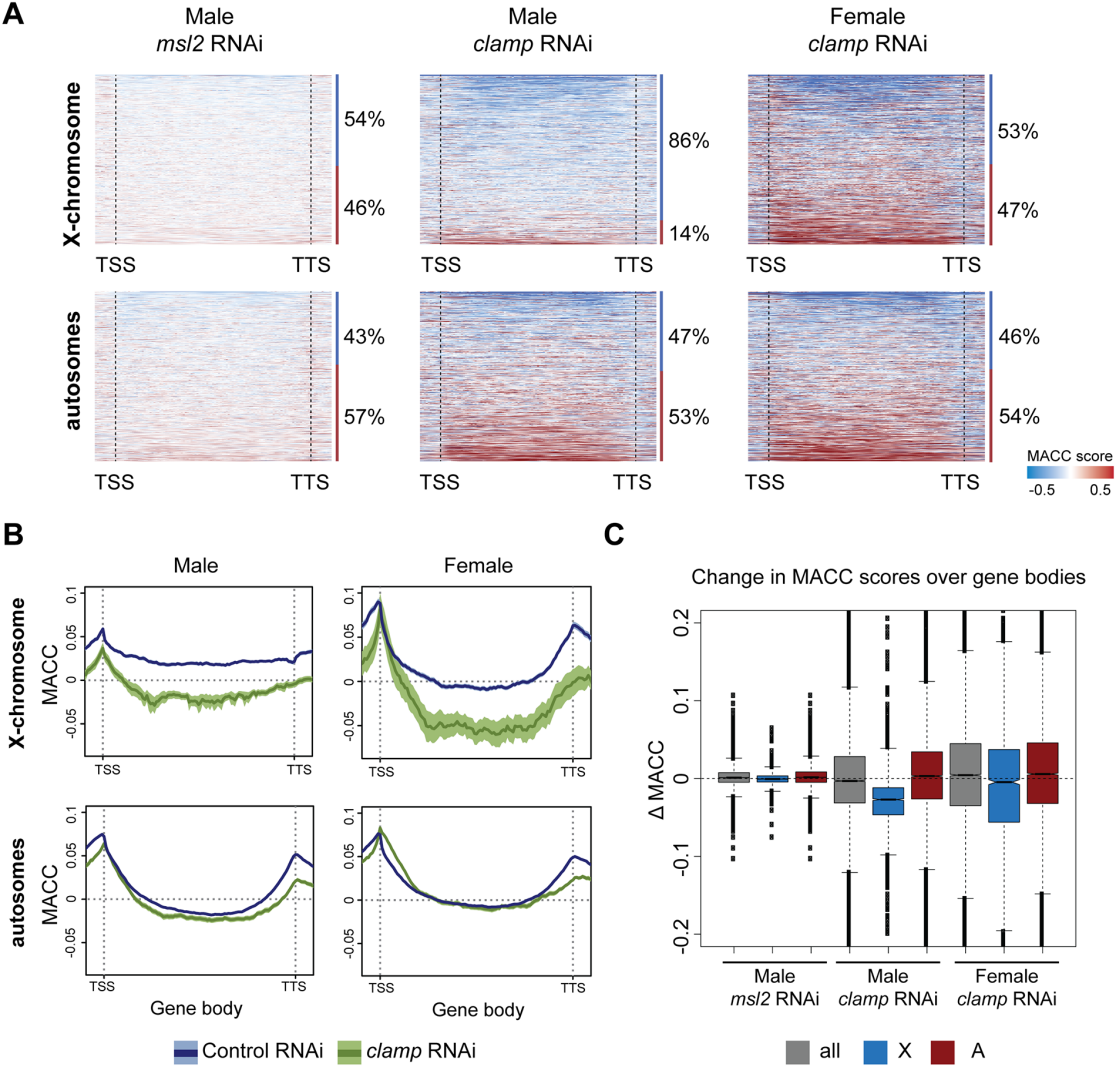
CLAMP directly affects accessibility of X-linked gene bodies in both males and females. A) The difference in MACC value (RNAi treatment—control) over gene bodies of all annotated genes was ranked by the magnitude of decrease (top) to increase (bottom) in accessibility. RNAi of *msl2* shows only a modest change in accessibility, whereas *clamp* RNAi results in accessibility changes in both males and females. Shown to the right of each heat map is the percent of genes that decrease (MACC<0, blue line) or increase (MACC>0, red line) in accessibility following the indicated RNAi treatment. B) Average MACC profiles across gene bodies are shown for male (S2) and female (Kc) cells separated by X-chromosomes and autosomes. X-linked gene bodies in both males and females have reduced accessibility after *clamp* RNAi treatment (green). The dark line represents the average MACC value, while 95% confidence intervals are represented by the lighter shading. C) The difference in MACC scores (Δ MACC) between control and RNAi over gene bodies was calculated. Plotted is the distribution of difference values for all chromosomes together (all), or separately for the X-chromosome and autosomes. Plotted is the median difference in MACC scores with the 95% confidence interval indicated by a notch around the median line.

To better define changes in accessibility between the control and RNAi treatments, we plotted the difference in accessibility between RNAi conditions (RNAi—control). These heat maps were rank-ordered based on the magnitude of accessibility change over the gene body following either *clamp* or *msl2* RNAi treatment (Fig 3A). We also determined the percentage of genes that either increase (RNAi—control > 0) or decrease (RNAi—control < 0) in accessibility (numbers shown next to the corresponding heat maps). We found only modest changes in chromatin accessibility on the X-chromosome and autosomes after *msl2* RNAi in males (Fig 3A and S4A Fig, *msl2* RNAi). In contrast, *clamp* RNAi resulted in a broad loss of chromatin accessibility over the majority (86%) of male X-linked genes (Fig 3A and S4A Fig, *clamp* RNAi), while male autosomes and female chromosomes exhibited nearly equal percentages of genes with increased vs. decreased accessibility (Fig 3A and S4A Fig).

Next, we analyzed the average effect of *clamp* RNAi over gene bodies after separating genes based on the enrichment of CLAMP within their TSS region. We determined groups by ranking CLAMP ChIP-seq enrichment from highest to lowest and dividing genes into two groups, “high” and “low” (see Methods). For genes highly enriched for CLAMP, *clamp* RNAi reduced chromatin accessibility over gene bodies on the X-chromosome more strongly than autosomes in both males and females (Fig 3B). This effect was specific to genes that are highly enriched for CLAMP because lowly enriched genes exhibited little difference in accessibility over gene bodies between the control and *clamp* RNAi treatments (S4B Fig). Taken together these results show that regions with a high enrichment of CLAMP have stronger changes in accessibility over gene bodies after *clamp* RNAi than those that are lowly enriched for CLAMP.

Consistent with the enrichment of CLAMP occupancy over X-linked gene bodies in both males and females [11], female cells exhibited an average X-specific decrease in accessibility over gene bodies that is similar to males (Fig 3B). Unlike male cells where 86% of X-linked genes decrease in accessibility following *clamp* RNAi, females have only a modestly larger percentage of genes with a decrease in accessibility compared to autosomes (Fig 3A, compare 53% to 46%). We asked how female cells could have a strong average X-specific reduction in accessibility after *clamp* RNAi when markedly fewer genes decrease in accessibility compared to males (compare 53% in females to 86% in males. To address this, we calculated the change in MACC scores along gene bodies between the control and RNAi treatments and plotted the distribution of these differences for all chromosomes combined, and the X-chromosome and autosomes separately (Fig 3C, see S1 Table for p-values). We found that genes on the female X-chromosome exhibited a stronger magnitude of decrease in chromatin accessibility over gene bodies compared to female autosomes. Therefore, the X-specific decrease seen on the average gene profiles in females (Fig 3B) is likely driven by a subset of X-linked genes (56%) that have a strong decrease in accessibility. The observation that CLAMP binds and alters chromatin accessibility more strongly on X-linked gene bodies than autosomal gene bodies in both males and females [9,11], suggests an MSL-independent function for CLAMP in promoting an open chromatin environment at a subset of X-linked gene bodies.

### CLAMP promotes chromatin accessibility at transcription start and termination sites genome-wide

**Fig 4:**
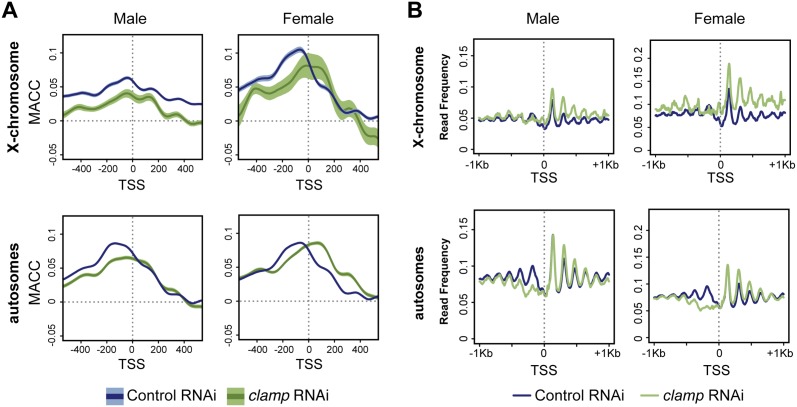
CLAMP promotes nucleosome positioning at 5’ends genome-wide. A) Average MACC profiles around transcription start sites (TSS) are shown for male (S2) and female (Kc) cells separated by X-chromosomes and autosomes. TSS at genes with highly bound CLAMP in both males and females are reduced in accessibility after *clamp* RNAi treatment (green) compared to control (blue). The dark line represents the average MACC value, while 95% confidence intervals are represented by the lighter colors. B) Average MNase-seq read frequency was calculated for the four MNase experiments and plotted +/- 1 Kb flanking annotated TSS. Upon *clamp* RNAi (green), there is a decrease in read frequency upstream of the TSS with a concurrent increase in occupancy within gene bodies.

In addition to gene bodies, CLAMP is highly enriched at TSS [9], where we observed CLAMP-mediated changes in accessibility at genes with the greatest CLAMP occupancy (S4A Fig). To investigate changes in accessibility around TSS in greater detail, we plotted average MACC values around TSS for genes with either a high or low enrichment of CLAMP defined as described as above (Fig 4). Following *clamp* RNAi, we observed a reduction in accessibility upstream of the TSS, approximately within the nucleosome-depleted region and the -1 nucleosome position, for genes where CLAMP is highly enriched independent of whether the genes are located on the X-chromosome or autosomes in males or females (Fig 4A). This change in accessibility after *clamp* RNAi was not observed at TSS with low CLAMP enrichment (S5A Fig). Further, these changes are specific to TSS because a randomized TSS control data set does not produce the same result (S5B Fig). We additionally observed some increase in accessibility immediately downstream of TSS in female cells after *clamp* RNAi treatment, which does not extend beyond 400 bp into the gene body (Fig 4A).

To further investigate this shift in the position of chromatin accessibility after *clamp* RNAi treatment, we generated average read frequency profiles around genes displaying the shift pattern. We selected 150 bp sized sequence fragments for our analysis under the assumption that these are enriched for mononucleosome-associated DNA. While these profiles largely represent nucleosome occupancy, MNase-seq data contain up to several percent of fragments associated with other chromatin-bound factors [13]. Importantly, such non-nucleosomal DNA fragments often originate from the regulatory loci including nucleosome-depleted regions at the TSS of active genes. Our results indicate that after *clamp* RNAi, there is a loss of precise positioning of occupancy peaks, specifically those that flank nucleosome-depleted region upstream of the TSS on both the X-chromosome and autosomes in both male and female cells (Fig 4B, S5C and S5D Fig). Furthermore, we observed the largest changes in read frequency using the lowest 1.5 Unit MNase concentration, indicating that the position of accessible nucleosomes and other chromatin-associated factors associated with accessible chromatin regions are most dependent on CLAMP (S5C and S5D Fig). Therefore, CLAMP functions to promote the specific localization of chromatin accessibility upstream of the TSS throughout the genome in both male and female cells.

In addition to TSS, we also investigated chromatin changes over TTS because CES are biased towards the 3’ ends of genes [12]. We observed CLAMP-mediated changes in accessibility at TTS for genes with the greatest CLAMP occupancy (S4A Fig). Similar to TSS and gene bodies, TTS exhibited a reduction in accessibility following *clamp* RNAi on the male X-chromosome (S5E Fig), which we also observed on the male autosomes, as well as all female chromosomes (S5E Fig). We conclude that like TSS, CLAMP promotes accessibility at TTS genome-wide.

### CLAMP differentially regulates transcription on the male X-chromosome compared with autosomes

To analyze the relationship between transcriptional and chromatin changes upon CLAMP depletion, we used the Start-seq technique [28]. The Start-seq technique specifically measures nascent RNAs produced by engaged RNA Polymerase II (Pol II) near transcription start sites, or Start-RNAs. Start-seq is a more precise measure of promoter Pol II activity than mRNA-seq, which also measures elongation, mRNA export, and stability. Using our Start-seq data, we redefined TSS based on the location where Pol II is engaged to generate a new subgroup of TSS called observed TSS (obsTSS) (S2 obsTSS: 9,740; Kc obsTSS: 10,067; TSS in FBv5.57: 13,903) [28]. We found that after *clamp* RNAi, approximately 10% of obsTSS were significantly changed (p<0.05) in their Start-RNA abundance in male cells, while approximately 7% were significantly changed in female cells (S2: 1,007 out of 9740: 10.3%; Kc: 680 out of 10,067: 6.8%).

**Fig 5:**
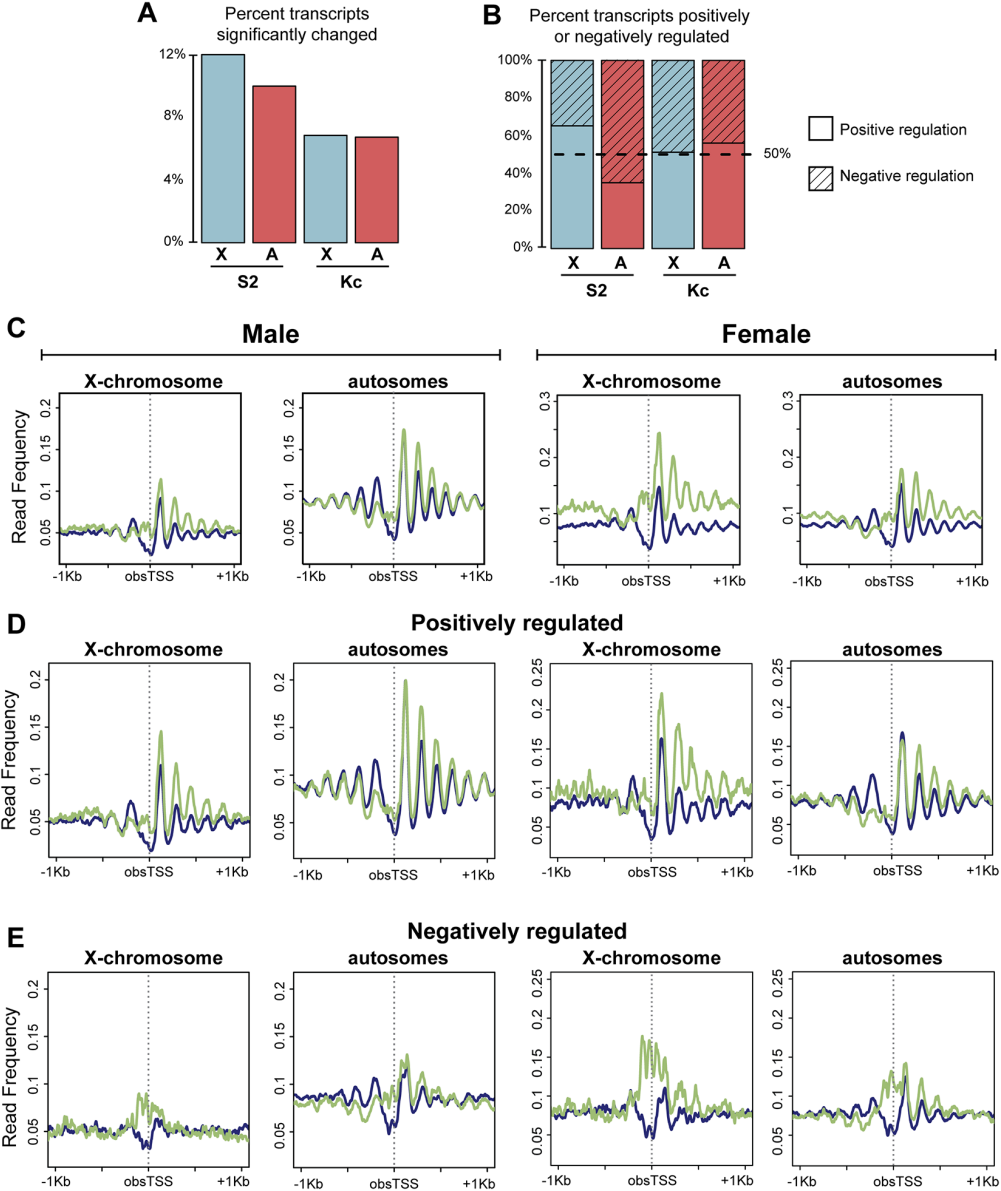
CLAMP functions to regulate transcription genome wide through regulating nucleosome positioning. A) Of the total number of transcripts aligned to the genome in males, approximately 12% (X-chromosome, blue) and 10% (autosomes, red) are significantly (p<0.05) changed following *clamp* RNAi treatment. The percentage of significantly changed transcripts in females is roughly equivalent for the X-chromosome and autosomes and is ~7%. B) The percent of significantly changed transcripts (p<0.05) that decrease in abundance after *clamp* RNAi (Positive regulation, un-hatched) or increase in abundance after *clamp* RNAi (Negative regulation, hatched) is plotted. CLAMP functions predominantly to promote transcription on the male X-chromosome, while on the male autosomes, it functions more frequently as a negative regulator. In females, CLAMP functions positively and negatively on all chromosomes at approximately equal frequencies. C) Average read frequency from all MNase titrations was plotted +/- 1 kb centered around obsTSS on the X and autosomes for genes with a CLAMP peak within +/- 200 bp of the obsTSS. There is a decrease in nucleosome positioning upstream of the TSS and an increase downstream of TSS on all chromosomes in both males (left) and females (right). D and E) X-chromosome and autosome obsTSS were categorized into quartiles based on the change in transcript abundance following *clamp* RNAi as measured by Start-seq. The two graphs on the left are for males while the graphs on the right are for females. Shown are average MNase-seq read frequency profiles for the quartiles with the largest decrease in transcription after *clamp* RNAi (D) (positively regulated) and largest increase in transcription after *clamp* RNAi (E) (negatively regulated).

We next determined the chromosomal distribution of significantly (p<0.05) changed Start-RNAs after *clamp* RNAi. We found that the significantly changed Start-RNAs were evenly distributed amongst all chromosomes in females, and modestly biased towards the X-chromosome in male cells, suggesting a role for CLAMP in transcriptional regulation on all chromosomes (Fig 5A and S5F Fig). For each chromosome, we then further examined the genes that displayed significantly increased (hatching) or decreased (no hatching) Start-RNA levels after *clamp* RNAi (Fig 5B and S5G Fig).

The male X-chromosome had the greatest percentage of transcripts positively regulated by CLAMP (62%), which was expected given the role of CLAMP in male X-chromosome dosage compensation which increases transcript levels (Fig 5B and S5G Fig). While transcription levels and chromatin accessibility tend to be highly correlated, we cannot fully determine whether the observed transcriptional changes following *clamp* RNAi are due to changes in accessibility or vice versa. However, in the case of the male X-chromosome, it is likely that changes in chromatin accessibility are separable from the transcriptional changes associated with MSL-dependent dosage compensation even if they are not separable from other transcription changes for the following reason: While MSL complex mediates the transcriptional changes associated with dosage compensation [20,22], it does not promote the same chromosome-wide changes in chromatin accessibility that are facilitated by CLAMP (Fig 1B and 1C) [7]. Furthermore, Start-seq measures transcription initiation and early elongation, which is less regulated by MSL complex than elongation over gene bodies [22,29]. Therefore, the CLAMP-mediated transcriptional changes observed are more likely to be linked to changes in chromatin accessibility than to a loss of dosage compensation.

In contrast to the male X-chromosome, CLAMP negatively regulates approximately 65% of genes on all autosomal arms with an even stronger effect on the heterochromatic 4^th^ chromosome (Fig 5B and S5G Fig). In females, CLAMP functions at approximately equal frequencies as both a positive and negative regulator of transcription on all chromosomes (Fig 5B and S5G Fig). Overall, CLAMP functions to promote gene activation more frequently on the male X-chromosome compared to male autosomes (Fig 5A and 5B).

To define how CLAMP-regulated transcriptional changes correlate with its role in chromatin accessibility, we compared average MNase-seq read frequencies over X-linked and autosomal genes that were either positively or negatively regulated by CLAMP as we did previously for TSS (Figs 4B and 5C). We analyzed genes that are highly bound by CLAMP as determined by ChIP-seq analysis and regulated by CLAMP as determined by Start-seq analysis (Fig 5) to define effects that are most likely to be directly mediated by CLAMP, presumably in combination with different co-factors within different genomic contexts. Genes that are positively regulated by CLAMP exhibited strong positioning over 5’ ends and gene bodies independently of genomic location (Fig 5D, S6A and S6B Fig). This pattern is similar to the pattern observed along the male X-chromosome (Fig 4B). In contrast, genes negatively regulated by CLAMP exhibited reduced nucleosome positioning after *clamp* RNAi treatment (Fig 5E, S6A and S6B Fig). For these genes, changes occur specifically over the nucleosome-depleted region at promoters and not along gene bodies.

To examine whether changes in MNase-seq read frequency after *clamp* RNAi differ when genes are expressed at different levels, we generated similar read frequency profiles for quartiles of genes ranked from lowest to highest expression level based on Start-seq read abundance in the control RNAi condition. Genes were further separated into additional classes based on whether they were located on the X-chromosome or autosome and if they were positively or negatively regulated by CLAMP (S7 and S8 Figs). In general, genes within the lowest three quartiles of gene expression exhibit similar changes in occupancy profiles after *clamp* RNAi. In contrast, the chromatin structural changes after *clamp* RNAi at genes in the top quartile are the strongest. We conclude that CLAMP regulates the chromatin organization of the most highly expressed genes more strongly than lowly expressed genes, with similar effects on the X-chromosome and autosomes. Overall, genes positively regulated by CLAMP exhibit changes in chromatin accessibility more similar to the average profile for genes on the male X-chromosome compared with those negatively regulated by CLAMP. In addition, we observed the same chromatin accessibility changes at CLAMP-regulated genes in both male and female cells, suggesting that the mechanisms by which CLAMP regulates chromatin and transcription at promoters are shared between the sexes.

### CLAMP promotes NURF recruitment to CES but not promoters in males

**Fig 6:**
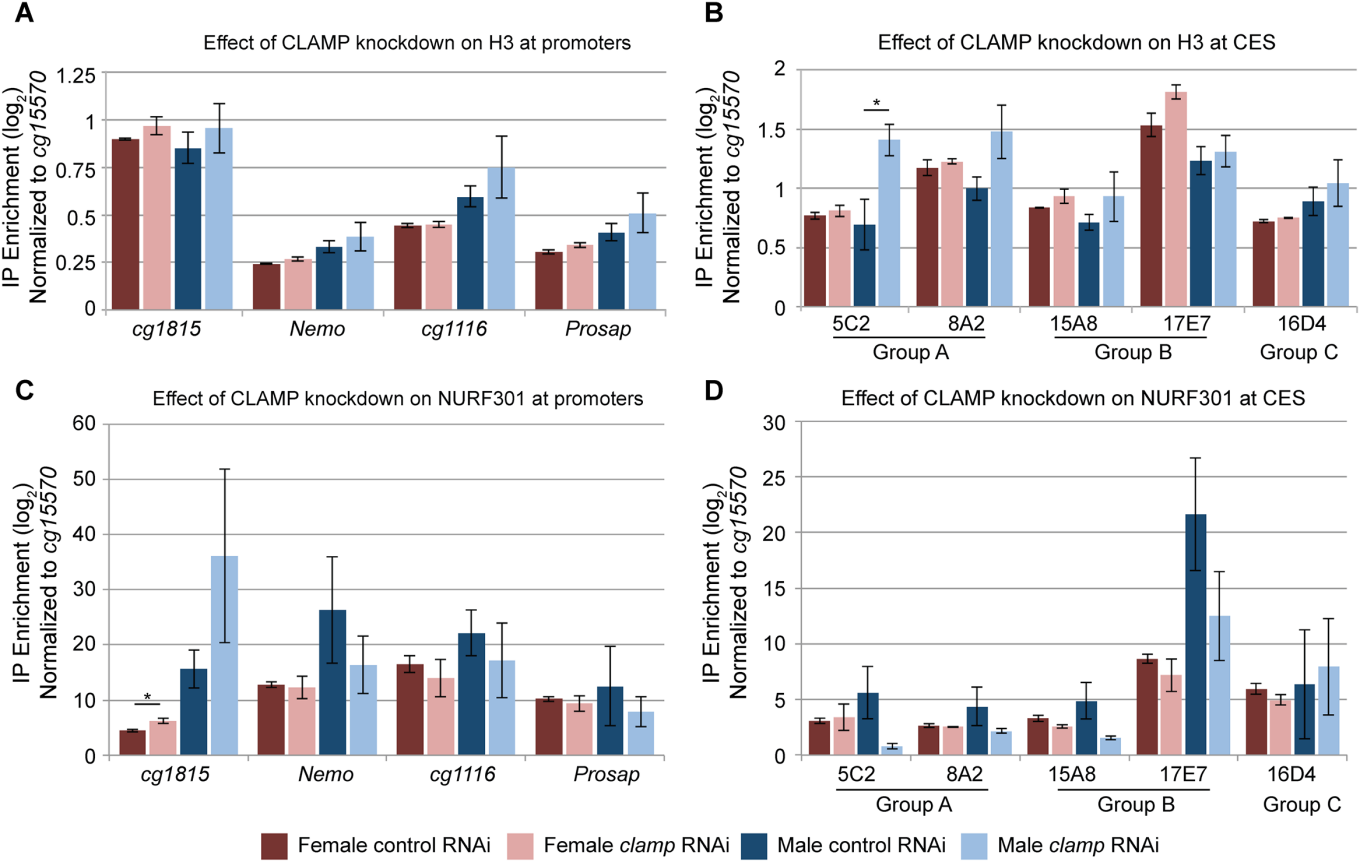
CLAMP promotes recruitment of NURF to CES, but not promoters. A) Chromatin Immunoprecipitation following by qRT-PCR was performed for Histone H3 at promoters of genes that are either positively (*cg1116* and *prosap*) or negatively (*cg1815* and *nemo*) regulated by CLAMP as measured by Start-seq. For all graphs, plotted is the average log_2_ fold enrichment value normalized to *cg15570*, a gene with no CLAMP enrichment with +/- 1 standard error of the mean (S.E.M). At all promoters tested there is no effect of *clamp* RNAi on H3 occupancy. B) H3 occupancy was measured by ChIP at five CES from the three subclasses. The CES with the greatest synergy between CLAMP and MSL complex (Group A) exhibit the strongest increase in H3 occupancy following *clamp* RNAi. The different in enrichment at CES5C2 was determined to be significant (* = p-value <0.05) by using a student’s T-test to compare differences between the *clamp* and control RNAi conditions. C) Enrichment of the NURF complex component NURF301 was measured at the same promoters as H3 occupancy. No significant differences were detected between the control and *clamp* RNAi treatments with the exception of *cg1815* in females (* = p-value <0.05). D) The effect of *clamp* RNAi on NURF occupancy was measured different subgroups of CES. There are observed reductions in NURF occupancy in males at CES within all groups with the exception of Group C CES.

Thus far, our analysis does not specifically distinguish between changes in chromatin accessibility that result from changes in nucleosome occupancy or occupancy of other factors that interact with chromatin. To measure changes in nucleosome occupancy, we performed Chromatin Immunoprecipitation followed by qRT-PCR (ChIP qRT-PCR) for the core Histone 3 (H3). We first measured the effect of *clamp* RNAi on H3 enrichment within the nucleosome-depleted region of four promoters differentially regulated by CLAMP at the level of chromatin accessibility and transcription (two positively regulated and two negatively regulated) (S9A and S9B Fig). We observed no statistically significant difference in H3 enrichment in either males or females between the control and *clamp* RNAi treatment at any of the tested promoters (Fig 6A, S2 Table). This suggests that regulation of accessibility at TSS by CLAMP does not involve changes in nucleosome occupancy. We therefore conclude that the mechanism by which CLAMP regulates accessibility within the upstream TSS region occurs independent of histone occupancy. Instead, accessibility may be regulated by the recruitment of other factors known to influence chromatin accessibility at TSS such as pausing factors like the Negative Elongation Factor (NELF) complex or even the Pol II complex itself [30–32].

We also measured H3 occupancy at CES to test whether changes in accessibility at these sites after *clamp* RNAi result from changes in nucleosome occupancy. We selected CES that exhibit CLAMP-dependent decreases in chromatin accessibility (S9B and S9C Fig). In contrast to candidate promoters, we found that *clamp* RNAi increases histone occupancy at the two-tested Group A CES in males, where CES5C2 was determined to exhibit the most significant change (p-value <0.05) (Fig 6B and S2 Table). Thus, CLAMP can regulate chromatin accessibility either by depleting histones or affecting interactions of non-histone factors with chromatin.

To define a possible mechanism by which CLAMP decreases histone occupancy at CES, we measured how CLAMP regulates recruitment of the Nucleosome Remodeling Factor (NURF) chromatin-remodeling complex. Several lines of evidence suggest a possible functional link between CLAMP and NURF. First, NURF has been previously implicated in regulating the chromatin organization of the male X-chromosome [33]. Second, CLAMP and the NURF301 chromatin remodeler both repress the expression of the male-specific non-coding *RNA on the X (roX)* RNAs in females [10,34]. Third, independent proteomic analyses of the MSL complex and dREAM chromatin-modifying complex identified both CLAMP and NURF301 as interacting factors [35,36]. Fourth, the well-studied transcription factor, GAGA Factor (GAF) shares a similar GA-rich binding motif with CLAMP and is known to promote the recruitment of the NURF complex [37–41]. Therefore, we investigated whether CLAMP recruits NURF to regulate the chromatin accessibility of the X-chromosome.

To test whether CLAMP promotes NURF recruitment to CES, we examined the same CES that we profiled for H3 occupancy and performed ChIP-qRT-PCR using an antibody specific for the NURF301 protein after control or *clamp* RNAi treatment. Co-occupancy of CLAMP and NURF301 was confirmed at each of these sites using previously published CLAMP ChIP-seq and NURF301 ChIP-chip data from the modENCODE project (S9 Fig) [42]. At all CES tested, we observed that CLAMP promotes NURF301 recruitment in males but not in females (Fig 6D and S2 Table), suggesting a potential mechanism for mediating changes in chromatin accessibility of the male X-chromosome. Because the ability of CLAMP to recruit NURF is specific to MSL-dependent Group A and B CES in males, it is possible that cooperation with MSL complex is important for this process. In contrast, *clamp* RNAi does not alter NURF occupancy at most promoters tested in males or females (Fig 6C) suggesting that mechanisms by which CLAMP alters chromatin accessibility at CES and TSS are different, comprising two distinct context-specific roles for the same protein. Overall, our data suggest that an X-chromosome enriched ubiquitous transcription factor that can act over long distances (CLAMP) and a sex-specific protein complex that acts locally (MSL complex) function together to generate a highly specialized chromatin domain that coordinates gene activation on the male X-chromosome.

## Discussion

Specifically identifying the X-chromosome to distinguish it from autosomes is the key initial step in dosage compensation across species. A specialized chromatin environment is present on the dosage compensated X-chromosome to allow it to be distinguished from autosomes and balance gene expression. However, the mechanism by which this specialized chromatin environment is established remained poorly understood. Here, we demonstrate that the non-sex-specific CLAMP protein functions to increase overall chromatin accessibility levels on the *D*. *melanogaster* male X-chromosome but not autosomes. We hypothesize that this chromosome-wide regulation results from clustered long GA-rich repeats within CES that increase CLAMP occupancy specifically on the X-chromosome [11]. CLAMP increases accessibility of chromatin over long genomic distances (~14 kb) surrounding its peak center (Fig 1E), suggesting a mechanism by which modest enrichment of the density of CLAMP binding sites promotes changes in chromatin accessibility across an entire chromosome. The ability of CLAMP to act at a kilo-base distance range may be linked to its presence in an insulator protein complex called the Late Boundary Complex (LBC) that was recently shown to function at CES that associate with each other in three-dimensions [7,24,43]. Because insulator complexes such as the LBC contribute to the overall three-dimensional organization of the genome [44], it is possible that the long-range chromatin effects we observe at CLAMP peaks are related to its role in the non-sex specific LBC.

In the early embryo, CLAMP transcript is maternally supplied [45], whereas expression of most MSL complex component-encoding transcripts does not occur until after activation of the zygotic genome [45]. It is possible that CLAMP functions early in development prior to MSL complex assembly to specifically open chromatin across the entire X-chromosome, priming it for MSL complex recruitment. In fact, the elevated density of CLAMP binding sites over gene bodies on the X-chromosome in both males and females [11] and the ability of CLAMP to function over long genomic distances independently of MSL complex (Fig 1E) supports this early function for CLAMP. Following the onset of zygotic transcription, MSL complex is assembled [46] and recruited to CES by CLAMP [9]. It is likely that CLAMP increases the local concentration of MSL complex at CES through opening chromatin and physically associating with the MSL complex [36]. In addition to CLAMP, direct binding of the MSL2 component to DNA through its CXC domain and Proline-rich domains at PionX sites [27] is important for MSL complex recruitment to CES. It is possible that MSL2 competes with CLAMP for the same GA-rich binding site or binds to a similar adjacent site within the clusters of GA-rich CLAMP binding sites that are present at CES. Once localized to CES, we hypothesize MSL complex opens chromatin locally (Fig 2), allowing for recruitment of additional CLAMP, generating a positive feedback loop [9] that results in the final open chromatin pattern along the entire male X-chromosome.

Interestingly, we found that CLAMP does not regulate accessibility at all CES similarly. For example, while CLAMP promotes chromatin accessibility at Group A CES, *clamp* RNAi results in enhanced accessibility at Group C CES in females and has no effect at these same sites in males. In females where dosage compensation does not occur it is possible that CLAMP interacts with additional effector complexes to prevent enhanced accessibility at Group C CES. In males, it is possible that CLAMP functions redundantly with other similar factors to assure that highly accessible locations such as the Group C CES remain open to promote dosage compensation. One candidate protein that may function redundantly or in competition with CLAMP is the well-studied GAGA transcription factor (GAF), a GA-repeat zinc finger protein with a similar domain structure and sequence recognition element to CLAMP [38]. Like CLAMP, GAF can also promote recruitment of the NURF301 protein complex [37–41]. We have recently determined that both CLAMP and GAF are members of the same LBC insulator complex [24], indicating that the two proteins can function cooperatively and possibly even redundantly. Future analysis of the relationship between CLAMP and GAF will allow us to better understand how their functional relationship modulates the recruitment of effector proteins.

CLAMP is a zinc finger protein that promotes the recruitment of different effector complexes that regulate chromatin accessibility including the MSL complex and NURF. Previous work has implicated the NURF chromatin remodeler complex in regulating the chromatin organization of the male X-chromosome [33]. Indeed, our results indicate that CLAMP promotes recruitment of the NURF chromatin-remodeling complex to a subset of the same CES where CLAMP occupancy is most influenced by MSL complex (Group A). We speculate that the synergy between CLAMP and MSL complex promotes NURF recruitment to a subset of CES where it contributes to the open chromatin environment by preventing the accumulation of nucleosomes.

In addition to regulating chromatin accessibility of the X-chromosome, CLAMP alters chromatin accessibility at TSS throughout the genome in both males and females. However, this occurs independently of NURF or changing histone occupancy levels at a subset of sites tested (Fig 6). At promoters, it is possible that CLAMP modifies chromatin accessibility through the recruitment of other factors known to promote the formation of open chromatin in this region. One potential factor is the Negative Elongation Factor (NELF) complex that regulates chromatin accessibility within the nucleosome-depleted region [47], and has been recently shown to interact with CLAMP (Urban *et al*., submitted). In this way, CLAMP-mediated changes in accessibility at TSS could be mediated by transcriptional regulation through a functional association between CLAMP and NELF. Future work will be required to examine the link between CLAMP and NELF occupancy genome-wide to determine if and how this relationship facilitates chromatin accessibility at transcription start sites.

While many transcription factors bind to thousands of locations throughout the genome, they often function differently depending on their genomic contexts [48–50]. For example, GAF performs diverse roles in gene regulation as well as chromatin accessibility. How does a single transcription factor regulate several distinct functions at different genomic locations in a context-specific manner? It is unlikely that most transcription factors are a part of a single stoichiometric complex. GAF, for example, functionally interacts with NURF [37,40], NELF [51,52], insulators [43,53], and the Polycomb complex [53–55]. CLAMP also functionally interacts with NELF and NURF, and is also a component of an insulator complex [24], making it plausible that CLAMP promotes diverse functions across the genome through associations with different effector complexes.

We suggest that CLAMP functions as an adapter protein that associates with different complexes at distinct genomic locations to perform diverse context-specific functions. Because CLAMP is a highly conserved [11] and maternally supplied protein, it is possible that CLAMP primarily functions as an adapter protein that over evolutionary time has been co-opted by different regulatory complexes to perform diverse functions. For example, as the GA-rich *cis*-elements that target CLAMP became enriched on the X-chromosome due to the activity of transposons [56], MSL complex likely evolved to interact with CLAMP because of its ability to enhance chromatin accessibility near active genes that require dosage compensation. Future analysis of the mechanism by which CLAMP interacts with specific effector complexes at different genomic locations will reveal how CLAMP performs its diverse context-specific roles. Overall, our work provides new insight into how a non-sex-specific factor can function together with a sex-specific complex to perform sex-specific and chromosome-specific functions that mediate specialized chromatin environments.

## Materials and methods

### Cell culture conditions

*Drosophila* S2 and Kc cells were maintained at 25°C in Gibco Schnieder’s *Drosophila* media (ThermoFisher Scientific) supplemented with 10% heat-inactivated Fetal Bovine Serum and 1.4X Antibiotic-Antimycotic (ThermoFisher Scientific). Cells were passaged every 2–3 days to maintain appropriate cell density.

### RNAi treatment of *Drosophila* cells

Generation of dsRNA targeting *gfp* (control), *clamp*, and *msl2* for RNAi has been previously validated and described [9,20,57]. In this study, the templates for *clamp* and *msl2* dsRNA were generated using PCR amplification from bacterial artificial chromosomes (BAC) available from the BACPAC resources program **(***clamp* = Ch322 20C06, *msl2* = Ch321 59O03) [58]. PCR products were used as templates to generate dsRNA with the T7 Megascript kit (Ambion, Inc.), followed by purification with the Qiagen RNeasy kit (Qiagen).

RNAi was performed in T75 tissue culture flasks. A total of 1.2x10^7^ S2 or Kc cells suspended in 6mLs of Gibco Schenieder’s *Drosophila* media without FBS were added to a T75 tissue culture flask containing 135μg *gfp*, *clamp*, *or msl2* dsRNA in 3mL ultra pure water. The cells were serum starved at room temperature for 45 minutes before adding 6mLs of Gibco Schneider’s *Drosophila* media supplemented with 10% FBS to the flask containing the dsRNA and cells. Cells were incubated for 6 days and upon collection efficiency of the RNAi treatment was determined before performing the desired experiment.

### Validation of RNAi efficiency

#### Sample preparation and Immuno-blotting of CLAMP

Following 6 days of RNAi treatment, cells were scraped and 500μL was collected for immuno-blotting. To extract protein, cells were first pelleted at 5,000xg for 3 minutes at 4°C. Cell pellets were next washed in 100μL 1X phosphate buffered saline before a second centrifugation. The supernatant was removed and the cell pellets were resuspended in 40μL of lysis buffer (50mM Tris-HCl pH 6.8, 150mM NaCl, 0.5% SDS, and 0.5X protease inhibitors (Roche)). After a 5-minute incubation at room temperature, the lysates were vortexed briefly. The samples were then cleared by centrifugation at room temperature at 14,000xg for 10 minutes. The supernatant was transferred to a new tube and the protein abundance was quantified using a Qubit Fluorometer (ThermoFisher Scientific).

To immuno-blot, a total of 5μg of protein was loaded on a pre-cast Tris Glycine gel (ThermoFisher Scientific) and immobilized on PVDF membrane using the iBlot transfer system (ThermoFisher Scientific). CLAMP (1:1000, rabbit, SDIX) and Actin (1:400,000, mouse, Millipore) proteins were detected using the Western Breeze kit (ThermoFisher Scientific) following the manufacturer’s instructions.

#### Sample preparation and quantitative Real-Time PCR for analysis of transcript abundance

To determine transcript abundance of *msl2* and *roX2* following RNAi treatment, 500μL of cells were collected following the 6-day incubation period. After pelleting cells and removing the media, total RNA was extracted using the RNeasy Plus RNA extraction kit (Qiagen). A total of 1μg of RNA was reverse-transcribed to cDNA using the SuperScript Vilo cDNA Synthesis kit (ThermoFisher Scientific) by following the manufacturer’s instructions. Targets were amplified from cDNA using validated primers at a concentration of 200nM. Primer sequences for qRT-PCR are available upon request. Three technical replicates for each sample were amplified using SYBR Green on an Applied Biosystems StepOnePlus^™^ Real-Time PCR System. The obtained relative abundance values were averaged and used to calculate ΔCt relative to PKA as an internal control.

### MNase-titration and sequencing

Preparation of two biological replicates of *Drosophila* S2 and Kc cells for RNAi treatment with *gfp*, *clamp*, or *msl2* was performed in T75 flasks. MNase titration was performed after 6 days of RNAi treatment as previously described [13].

### Start-sequencing sample preparation and analysis

Start-sequencing was performed from three biological replicates of S2 and Kc cells that were treated for 6 days with either *gfp* (control) or *clamp* dsRNA. RNAi for Start-seq was performed as described above for MNase-seq with the exception that T150 flasks were used instead of T75. This resulted in a doubling of all reagents to perform the RNAi treatment. Total RNA was extracted from nuclei using Trizol reagent (Invitrogen) and transcription start site RNAs were isolated and prepared for sequencing as previously described [28]. Analysis for Start-seq was performed as previously described [28].

### Chromatin immunoprecipitation of NURF301 and H3

Chromatin was prepared from *gfp* (control) and *clamp* dsRNA treated cells prepared in T150 flasks after 6 days of incubation. To prepare for chromatin immunoprecipitaton, cells were first scraped from the bottom of the flask to resuspend adhered cells. Formaldehyde (37% stock) was added to a final concentration of 1% and the cells were crosslinked for 10 minutes by shaking at room temperature at 110 rpm. The crosslinking reaction was quenched with the addition of 2.5M glycine to a final concentration of 125mM. Fixed cells were transferred to a 15mL falcon tube and placed on ice.

Following crosslinking and quenching, the media containing formaldehyde and glycine was removed after pelleting cells by centrifugation at 4°C for 5 minutes at 1,500xg. The cells were washed briefly by resuspending in 15mL of PBS-EDTA (1X PBS, 0.5mM EDTA pH 8.0, 0.2mM PMSF). Cells were pelleted as before and the supernatant removed. Next, the cells were resuspended in 6mL of ChIP wash A (10mM HEPES pH 7.6, 10mM EDTA pH 8.0, 0.5mM EGTA pH 8.0, 0.25% Triton X-100, 1X PI, 0.2mM PMSF, filter sterilized) and rotated for 10 minutes at 4°C. After rotation, the cells were pelleted once again and the supernatant removed. The cell pellet was then resuspended in 6mL ChIP wash B (10mM HEPES pH 7.6, 100mM NaCl, 1mM EDTA pH 8.0, 0.5mM EGTA pH 8.0, 0.01% Triton X-100, 1X PI, 0.2mM PMSF, filter sterilized). The cells were rotated for 5 minutes at 4°C. A small aliquot was removed to determine total cell quantity using Trypan Blue (ThermoFisher Scientific). After washing the cells in ChIP wash B, the cells were pelleted flash frozen in liquid nitrogen for storage at -80°C.

Using the total cell count, cell pellets were resuspended in an appropriate amount of lysis buffer (0.1% SDS, 50mM Tris-HCL pH 8.0, 10mM EDTA pH 8.0) to obtain a cell concentration of 1x10^8^ cells/mL. The cells were lysed for 10 minutes by rotating at 4°C before aliquoting equal volumes (between 100-300uL) into Protein LoBind Safelock tubes (Eppendorf). The cell lysates were then sonicated for 3 cycles totaling 5 minutes each on high setting using a water bath sonicator (Bioruptor, Diagenode) programmed to pulse 30 sec on, 30 sec off. After sonication, replicate samples were pooled and centrifuged at 4°C for 10 minutes at a speed of 13,000 rpm to pellet the insoluble chromatin. Solubilized chromatin was transferred to a 15mL falcon tube, to which 9 volumes of ChIP dilution buffer was added (0.01% SDS, 16.7mM Tris-HCL pH8.0, 1.2mM EDTA pH 8.0, 1.1% Triton X-100, 167mM NaCl). Diluted chromatin was filtered on a PolyPrep chromatography column (BioRad) before aliquoting 1mL into Protein LoBind safelock tubes. Additionally, 400uL was removed for Input DNA. Chromatin aliquots were flash frozen for storage at -80°C until after Input was checked for chromatin size distribution.

#### Cleanup of Input DNA

In order to determine the size distribution of chromatin fragments, the 400uL of Input DNA was reverse crosslinked by incubation overnight at 65°C after the addition of 21.5uL 20% SDS (final concentration 1%), 15uL of 5M NaCl (final concentration 170mM NaCl) and 1uL of RNase A (Roche). Next, an additional 1uL of RNase A was added for 30 minutes at an incubation temperature of 37°C. After this, protein was digested from the chromatin by adding 20uL of 1M Tris-HCL pH 6.8 (final concentration 42mM Tris-HCL), 10uL EDTA pH 8.0 (final concentration 10mM EDTA), and 3uL 20mg/mL Proteinase K (final concentration 0.13 mg/mL Proteinase K) and incubating for 90 minutes at 45°C. Following protein digestion, a standard phenol-chloroform extraction was performed followed by ethanol precipitation. DNA pellets were resuspended in 100uL ultra pure water and quantified. To determine fragmentation distribution was between 100-500bp, 200ng of chromatin was visualized on a 2% agarose gel.

#### Immunoprecipitation and DNA cleanup

To begin the immunprecipitation, either 2.5ug of NURF-301 antibody (Novus Biologicals #40360002) or 2ug of Histone H3 (Abcam #ab1791) was added to 1mL of previously prepared chromatin and rotated overnight at 4°C. The next day, 60uL of salmon sperm blocked Protein A agarose beads (Millipore) were added and the samples rotated for an additional 2 hours at 4_C. After incubation, the beads were pelleted by centrifugation at 4°C for 3 minutes at 1,000xg. The beads were washed a total of 6 times in a series of wash solutions. For each wash, first the beads were resuspended in 1mL solution, then rotated for 5 minutes at 4°C. Beads were then pelleted by centrifugation at 1,000xg for 3 minutes at 4°C, the supernatant was removed, and the beads resuspended in the next wash solution. The washes include: 1.) two washes in RIPA 150 Buffer (50mM Tris-HCL pH 8.0, 1% NP-40, 2mM EDTA pH 8.0, 0.1% Sodium Deoxycholate, 0.1% SDS, 150mM NaCl, 1X protease inhibitors, 0.2mM PMSF, 1mM DTT, filter sterilized); 2.) one wash in RIPA 300 buffer (50mM Tris-HCL pH8.0, 1% NP-40, 2mM EDTA pH 8.0, 0.1% Sodium Deoxycholate, 0.1% SDS, 300mM NaCl, filter sterilized), 3.) one wash in LiCl/TE buffer (0.25M LiCl, 1% NP-40, 1% Sodium Deoxycholate, 10mM Tris-HCL pH 8.0, 1mM EDTA pH 8.0, filter sterilized), and 4.) two washes in TE buffer (10mM Tris-HCL pH 8.0, 1mM EDTA pH 8.0, 0.01% SDS, 1X protease inhibitors, 0.2mM PMSF, filter sterilized). After the final TE buffer wash, the beads were eluted twice in 250uL sodium bicarbonate Elution Buffer (1% SDS, 0.1M Sodium Bicarbonate) by rotating at room temperature for 15 minutes and pelleting by centrifugation at room temperature for 3 minutes at 1,000xg to collect the eluate. The 500uL of immunoprecipitated samples were reversecrosslinked overnight at 65°C after the addition of 20uL 5M NaCl (final concentration 200mM NaCl). After reverse crosslinking the immunoprecipitated samples overnight, 1uL of RNase A (Roche) was added and the samples incubated for 30 minutes at 37°C. Next, 20uL 1M Tris-HCL pH 6.8 (final 40mM Tris-HCL), 10uL 0.5M EDTA pH 8.0 (final concentration 10mM EDTA), and 3uL 20mg/mL Proteinase K were added to the samples and incubated for 90 minutes at 42°C. After digesting proteins, the DNA from the immunprecipiated samples was cleaned by using a standard phenol:chloroform extraction followed by ethanol precipitation. The immunoprecipitated DNA pellet was then resuspended in 100uL ultra pure water for downstream analysis by qRT-PCR.

### Quantitative Real-Time PCR for analysis of enrichment

To determine enrichment of NURF301 or H3 to chromatin entry sites and promoters, three independent biological ChIP replicates were performed. Targets were amplified from 2uL of Input (1%) and immunoprecipitated DNA using validated primers at a concentration of 200nM. Primer sequences for qRT-PCR are in S3 Table. Two technical replicates for each sample were amplified using SYBR Green on an Applied Biosystems StepOnePlus^™^ Real-Time PCR System. The obtained relative abundance values were then averaged and used for calculation of enrichment.

Internal normalization was performed using primers located within the *cg15570* control gene, which is a genomic locus unbound by CLAMP as determined by ChIP-seq. Internally normalized values were then normalized to the 1% Input sample to determine log_2_-fold enrichment of the immunoprecipitated sample compared to Input. Subsequently, the enrichment values from the three biological replicates were averaged and standard error of the mean was calculated.

### Datasets

CLAMP ChIP-seq data and MSL CES coordinates were taken from GSE39271. To determine overlap between CLAMP and NURF301, browser tracks were taken from GSE20829 (S2 cells) and GSE32845 (Kc cells). Data from this study is deposited to NCBI GEO with GEO number GSE99894.

### Data analysis

#### Chromatin accessibility (MACC) evaluation

Chromatin accessibility was evaluated as described previously [13]. In short, sequenced reads were aligned to the *D*. *melanogaster* reference genome (dm3) using Bowtie aligner [59]. Genomic positions with abnormally high numbers of mapped reads (*Z*-score = 7) were identified, and the tags mapped to such positions were discarded [60]. Frequencies of the mapped reads were computed in 100bp non-overlapping bins and normalized for the library size. Slopes of linear regression lines fitted on the normalized read frequencies obtained for each titration point (1.5U, 6.25U, 25U, and 100U MNase concentrations) were calculated for each bin. Log-scale was used for the MNase concentrations in the fitting procedure. The GC-content correction was applied to obtain the final accessibility scores (MACC values). Accessibility estimations were further validated with the following analyses: 1) to rule out a possible cross-sample bias, we confirmed that the average MACC profiles around a random set of genomic locations are similar for different RNAi conditions (S5B Fig). We also confirmed our main observation that the loss of chromatin accessibility on the male X-chromosome specifically occurs after *clamp* RNAi treatment is reproduced when MACC values were median-shifted to zero for each sample independently (S2F Fig). Finally, to rule out a potential bias due to different numbers of X-chromosomes and autosomes in the male cells, we recomputed MACC values counting each X-linked read twice and confirmed the CLAMP-specificity of the change in chromatin accessibility on male X-chromosome (S2G Fig).

#### Analysis of MACC profiles and estimation of statistical significance

Gene coordinates were taken according to the dm3 annotation. The modENCODE annotation of the enhancers that are active in S2 cells were used in the analysis [61]. TSS (TTS) proximal regions used in Fig 1 were defined as 500 bp upstream (downstream) of the gene start (end). Enhancer regions in the same analysis were defined as loci +/-500 bp around reported enhancer centers. The profiles around specified sets of sites were computed by using linear interpolation of MACC or read frequency values associated with 100-bp bins. The resulting average profiles were additionally smoothed in the 10-bp running window. The TSS proximal regions were defined as those within 1kb of gene starts. TSS-proximal regions overlapping with other genes were excluded from consideration. Assessment of statistical significance and other analyses were performed in R programming environment (http://r-project.org). Significance of accessibility or transcription change was calculated in R using the ‘limma’ package. Mann-Whitney test was used to estimate the significance of the observed effects.

## Supporting information

S1 Fig A) An anti-CLAMP and anti-Actin western blot was performed to confirm efficient reduction of CLAMP (62 kDa). Actin (42 kDa) is used as a loading control. B) Transcript abundance of *msl2* was tested using qRT-PCR to validate efficiency of the RNAi treatment. Transcript levels of *msl2* were reduced to levels similar to that in females following *msl2* RNAi in males. Error bars for transcript abundance represent +/- 1 Standard Error of the Mean (S.E.M.). C) Transcript abundance of *roX2* following *msl2* RNAi was measured as a functional test for efficiency of the RNAi treatment. Following *msl2* RNAi treatment, *roX2* abundance was significantly reduced in male cells, indicating functional reduction of MSL complex.
(PDF)

S2 Fig A) The distribution of MACC values from all experimental conditions shows no effect on overall MACC scores after *msl2* RNAi in males compared to control. In both males and females, treatment with *clamp* RNAi results in an overall decrease in MACC values. For all box and whisker plots, the median MACC value is plotted with the notch at the median line representing the 95% confidence interval. B) The distribution of MACC values from all experimental conditions is shown as in A for each of the replicates separately. MACC scores from the replicates are in strong agreement. C and D) The overall distribution of MACC scores is shown separately for each replicate for the X-chromosome (blue) and autosomes (red) of Control (*gfp*), *clamp* and *msl2* RNAi treated male (C) and female (D) cells. E) The difference in MACC value (Δ MACC) between control and RNAi treatment for an individual locus on either the X-chromosome or autosomes was calculated for both replicates separately. In males, the change in MACC scores indicates a reduction in X-chromosome accessibility following *clamp* RNAi but not *msl2* RNAi. F) Analysis of the per-bin pair-wise difference in the chromatin accessibility (MACC) between RNAi conditions. The computed MACC valued were additionally median-shifted to zero in each sample independently prior to cross-sample comparison. G) To compensate for different number of X and autosomes, the reads that aligned to the X-chromosome were counted twice. The MACC values were additionally median-shifted to zero as in (F).
(PDF)

S3 Fig A) Accessibility changes for different classes of genomic regions were normalized by the percentage of the genome covered by each feature. A change in accessibility was classified as either within a gene body (blue), at TSS/TTS (red), at an enhancer (green), or unannotated (purple). B) CLAMP ChIP-seq peaks [9] were separated into quartiles of increasing CLAMP occupancy, Q1 being the lowest enrichment and Q4 the highest. The corresponding MACC values for each quartile were plotted. Also shown are regions where there is no CLAMP peak (no peak). In general, regions enriched with CLAMP are more accessible independent of chromosomal location or sex. C) The distribution of MACC scores around CES obtained from randomized MACC scores in non-repetitive regions are shown for male (S2) cells. The darker line represents the average MACC value, while the lighter shading indicates the 95% confidence intervals. D) The distribution of MACC values in males after control (blue), *clamp* (green), and *msl2* (purple) RNAi are plotted centered on PionX sites.
(PDF)

S4 Fig A) Heatmaps of MACC scores over gene bodies are shown for all annotated genes upon control, *clamp* or *msl2* RNAi in male (S2) cells, and *clamp* RNAi in female (Kc) cells. Below, the difference in accessibility between control and RNAi treatment on the X-chromosome and autosomes is shown in the second row. Each heat map is rank-ordered by the level of CLAMP enrichment from ChIP-seq occupancy (shown on the left in green). B) Average MACC profiles along gene bodies are shown for male and female cells separated into X-chromosome and autosome plots. Shown are MACC profiles for genes that are lowly enriched for CLAMP with *clamp* RNAi treatment in green and control in blue. The dark line represents the average MACC value, while 95% confidence intervals are represented by the lighter colors.
(PDF)

S5 Fig A) Average MACC profiles for genes with low enrichment of CLAMP are shown centered on transcription start sites (TSS) and separated into X-chromosome and autosome plots for male and female cells. RNAi treatment of *clamp* (green) and control (blue) is indicated, where the dark line represents the average MACC value, while 95% confidence intervals are represented by the lighter colors. B) The distribution of average MACC values around randomized TSS in non-repetitive regions is plotted for male (S2) cells. The darker line represents the average MACC value and the lighter shading indicates the 95% confidence interval. C and D) The nucleosome read counts obtained for each concentration of MNase are shown under control (blue) and *clamp* (green) RNAi conditions and centered over annotated TSS. Each concentration is shown as a gradient color of the RNAi treatment. Nucleosome profiles are shown for the male X-chromosome and autosomes separately for both males (C) and females (D). E) Average MACC values (darker line) were plotted +/- 500bp centered on transcription termination sites (TTS) separated by the X-chromosome and autosomes in males and females. There is a reduction in accessibility after *clamp* RNAi (green) compared to the control (blue). The lighter shading surrounding the mean line on all plots represents the 95% confidence interval. F) The percentages of significantly changed transcripts (p<0.05) in males (left) and females (right) are shown for each chromosomal arm. The blue bar indicates the X-chromosome and autosomes are in red/pink. G) The percentages of significantly changed transcripts (p<0.05) that decrease in abundance after *clamp* RNAi (un-hatched) or increase in abundance after *clamp* RNAi (hatched) are shown for each chromosomal arm in males (left) and females (right).
(PDF)

S6 Fig A and B) Average MNase-seq read frequency profiles +/- 1 kb centered around obsTSS on the X and autosomes were generated for genes with a CLAMP peak within +/- 200 bp of the obsTSS. Profiles were generated for both males (A) and females (B). X-chromosome and autosome obsTSS were categorized into quartiles based on the ability of CLAMP to positively or negatively regulate transcription as measured by Start-seq. Shown are the profiles for each quartile.
(PDF)

S7 Fig Average MNase-seq read frequency profiles centered +/- 1 kb around obsTSS were generated for genes with a CLAMP peak within +/- 200 bp of the obsTSS. X-chromosome and autosome obsTSS were categorized into quartiles of increasing expression level as determined by transcript abundance in the control RNAi condition and separated by whether they are positively or negatively regulated by CLAMP.
(PDF)

S8 Fig Average MNase-seq read frequency profiles centered +/- 1 kb around obsTSS were generated for genes with a CLAMP peak within +/- 200 bp of the obsTSS. X-chromosome and autosome obsTSS were categorized into quartiles of increasing expression level as determined by transcript abundance in the control RNAi condition and separated by whether they are positively or negatively regulated by CLAMP.
(PDF)

S9 Fig Four promoter regions (A and B) and five CES (C and D) were tested for NURF301 recruitment in males and females. For each, enrichment for NURF301 (orange) and CLAMP (green) is shown. The MACC values after control, *clamp*, and *msl2* RNAi treatment are shown in blue where dark blue indicates positive values and light blue are negative. The average number of sequencing reads from the four MNase-seq experiments generated a nucleosome profile that is shown in purple for Control, *clamp*, and *msl2* RNAi. NURF301 recruitment was tested following *clamp* RNAi treatment by ChIP qRT-PCR at four promoters in males (A) and females (B), where the red bar underneath the gene is scaled to 100 bp. Similarly five CES were tested in males (C) and females (D). The black bar underneath the red CES bar indicates the location of the qRT-PCR product for the ChIP qPCR experiments.
(PDF)

S1 Table MACC score p-values. (PDF)

S2 Table Chromatin immunoprecipitation p-values for NURF301 and H3. (PDF)

S3 Table Primers sequences used in this study. (PDF)
