# Supplementary figures and images for: Enhanced chromatin accessibility of the dosage compensated Drosophila male X-chromosome requires the CLAMP zinc finger protein
Source: PLoS One. 2017 Oct 27;12(10):e0186855. doi: 10.1371/journal.pone.0186855 (PMC5659772; doi:10.1371/journal.pone.0186855)

**A**

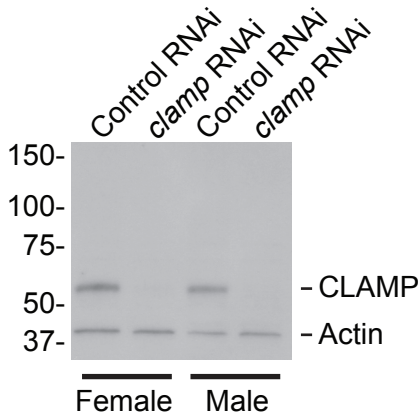

**B**

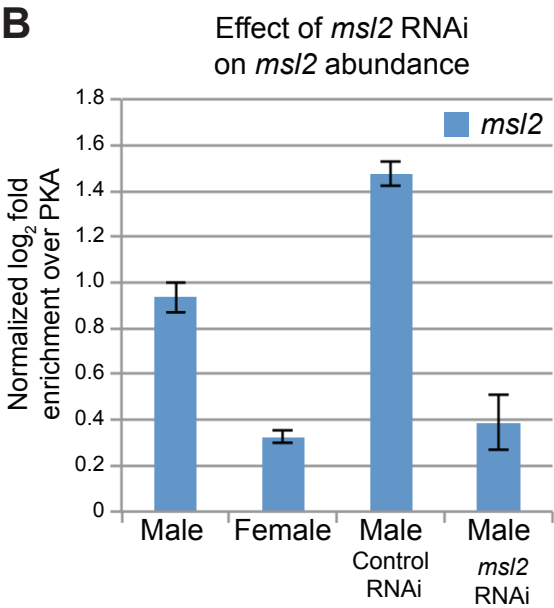

**C**

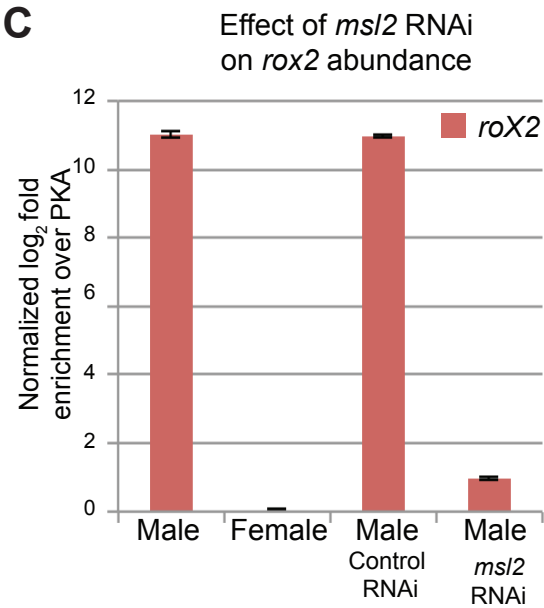

Supplement: S1 Fig — A) An anti-CLAMP and anti-Actin western blot was performed to confirm efficient reduction of CLAMP (62 kDa). Actin (42 kDa) is used as a loading control. B) Transcript abundance of msl2 was tested using qRT-PCR to validate efficiency of the RNAi treatment. Transcript levels of msl2 were reduced to levels similar to that in females following msl2 RNAi in males. Error bars for transcript abundance represent +/- 1 Standard Error of the Mean (S.E.M.). C) Transcript abundance of roX2 following msl2 RNAi was measured as a functional test for efficiency of the RNAi treatment. Following msl2 RNAi treatment, roX2 abundance was significantly reduced in male cells, indicating functional reduction of MSL complex. (PDF) [file pone.0186855.s001.pdf]

## S2 Fig (Urban, Kuzu)

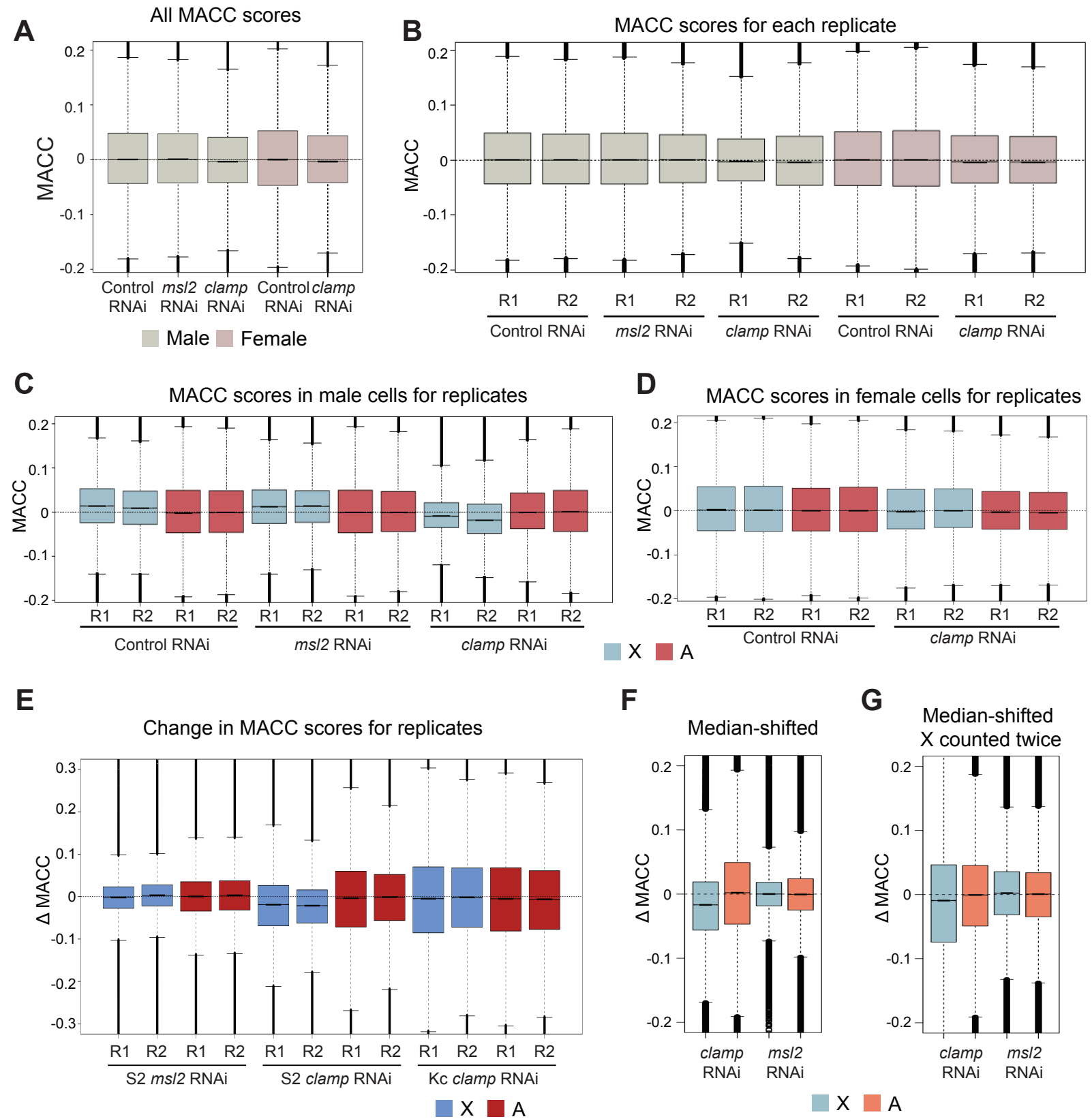

Supplement: S2 Fig — A) The distribution of MACC values from all experimental conditions shows no effect on overall MACC scores after msl2 RNAi in males compared to control. In both males and females, treatment with clamp RNAi results in an overall decrease in MACC values. For all box and whisker plots, the median MACC value is plotted with the notch at the median line representing the 95% confidence interval. B) The distribution of MACC values from all experimental conditions is shown as in A for each of the replicates separately. MACC scores from the replicates are in strong agreement. C and D) The overall distribution of MACC scores is shown separately for each replicate for the X-chromosome (blue) and autosomes (red) of Control (gfp), clamp and msl2 RNAi treated male (C) and female (D) cells. E) The difference in MACC value (Δ MACC) between control and RNAi treatment for an individual locus on either the X-chromosome or autosomes was calculated for both replicates separately. In males, the change in MACC scores indicates a reduction in X-chromosome accessibility following clamp RNAi but not msl2 RNAi. F) Analysis of the per-bin pair-wise difference in the chromatin accessibility (MACC) between RNAi conditions. The computed MACC valued were additionally median-shifted to zero in each sample independently prior to cross-sample comparison. G) To compensate for different number of X and autosomes, the reads that aligned to the X-chromosome were counted twice. The MACC values were additionally median-shifted to zero as in (F). (PDF) [file pone.0186855.s002.pdf]

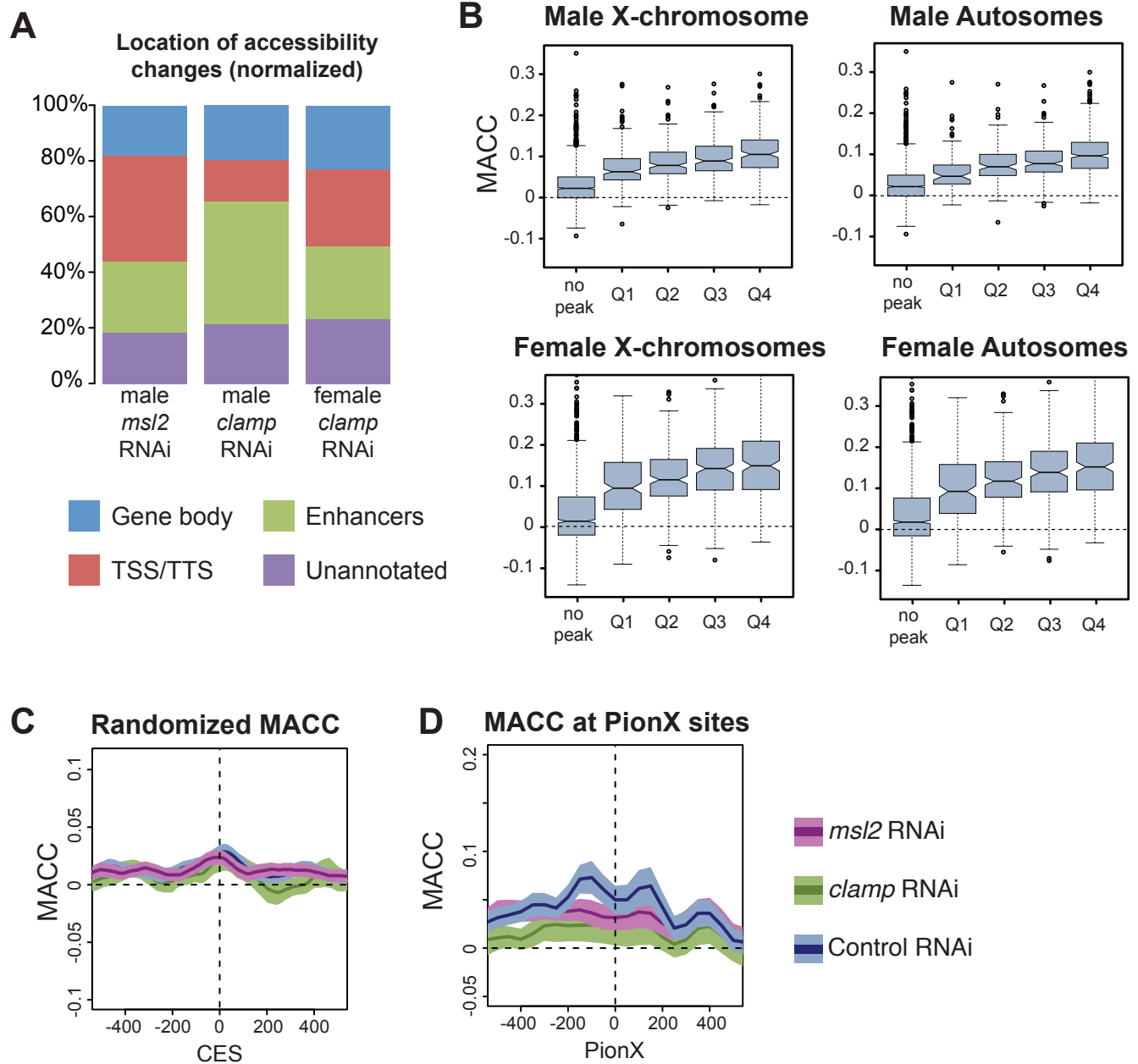

Supplement: S3 Fig — A) Accessibility changes for different classes of genomic regions were normalized by the percentage of the genome covered by each feature. A change in accessibility was classified as either within a gene body (blue), at TSS/TTS (red), at an enhancer (green), or unannotated (purple). B) CLAMP ChIP-seq peaks [9] were separated into quartiles of increasing CLAMP occupancy, Q1 being the lowest enrichment and Q4 the highest. The corresponding MACC values for each quartile were plotted. Also shown are regions where there is no CLAMP peak (no peak). In general, regions enriched with CLAMP are more accessible independent of chromosomal location or sex. C) The distribution of MACC scores around CES obtained from randomized MACC scores in non-repetitive regions are shown for male (S2) cells. The darker line represents the average MACC value, while the lighter shading indicates the 95% confidence intervals. D) The distribution of MACC values in males after control (blue), clamp (green), and msl2 (purple) RNAi are plotted centered on PionX sites. (PDF) [file pone.0186855.s003.pdf]

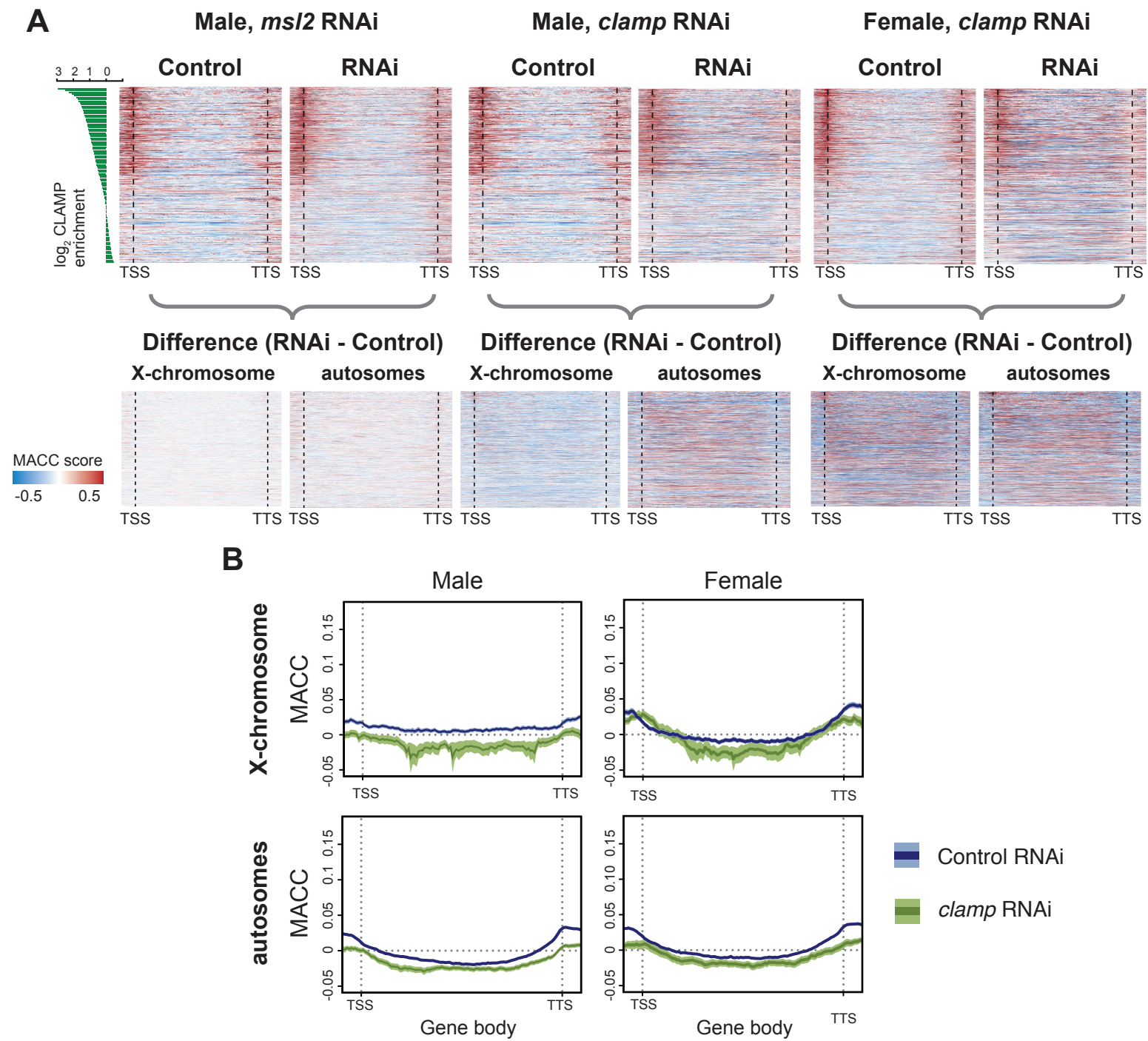

Supplement: S4 Fig — A) Heatmaps of MACC scores over gene bodies are shown for all annotated genes upon control, clamp or msl2 RNAi in male (S2) cells, and clamp RNAi in female (Kc) cells. Below, the difference in accessibility between control and RNAi treatment on the X-chromosome and autosomes is shown in the second row. Each heat map is rank-ordered by the level of CLAMP enrichment from ChIP-seq occupancy (shown on the left in green). B) Average MACC profiles along gene bodies are shown for male and female cells separated into X-chromosome and autosome plots. Shown are MACC profiles for genes that are lowly enriched for CLAMP with clamp RNAi treatment in green and control in blue. The dark line represents the average MACC value, while 95% confidence intervals are represented by the lighter colors. (PDF) [file pone.0186855.s004.pdf]

**A**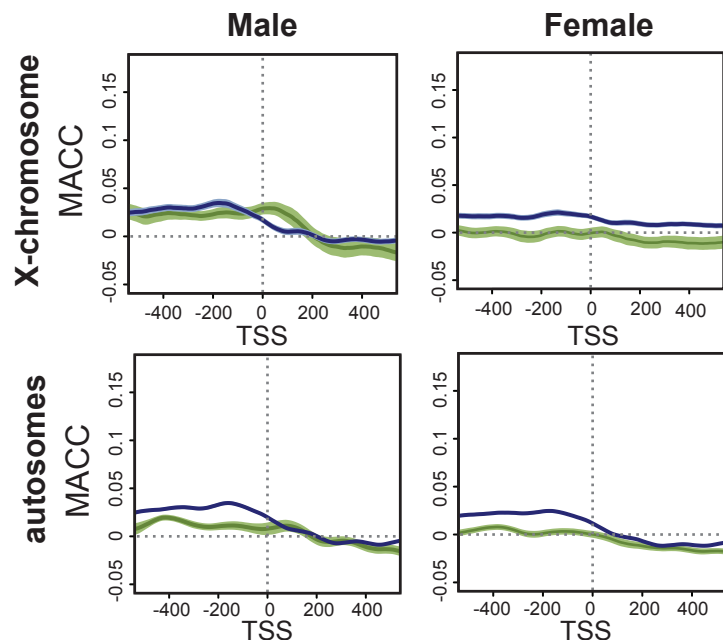**B**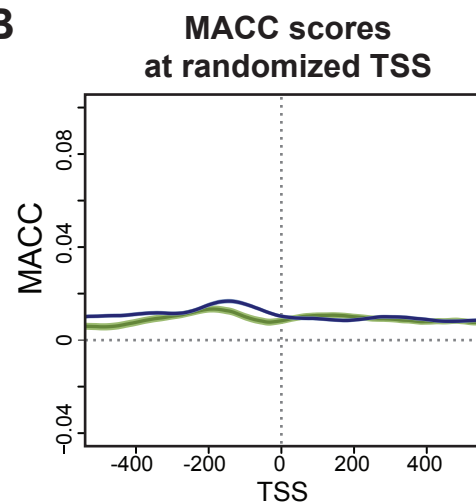**C**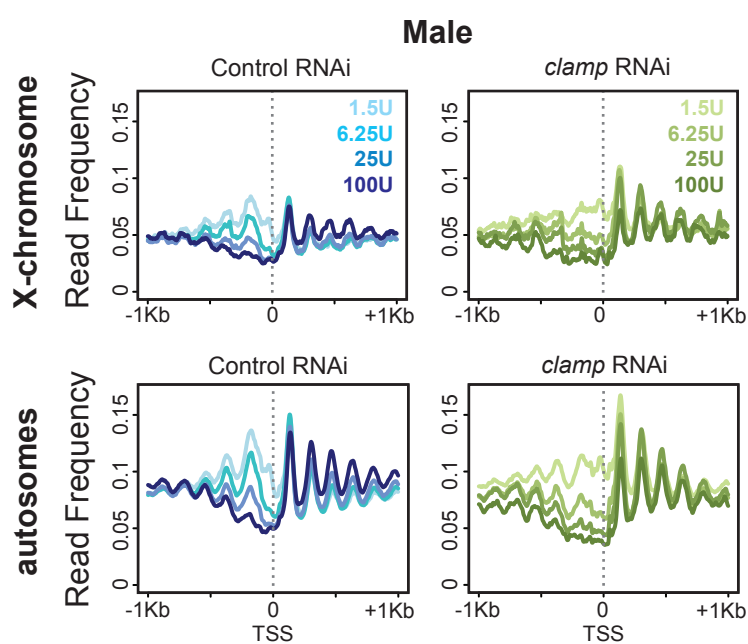**D**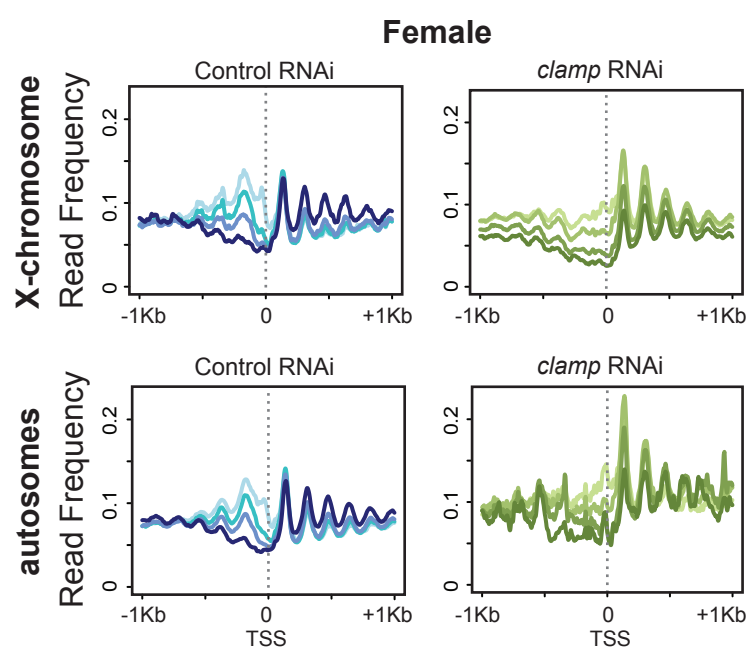**E**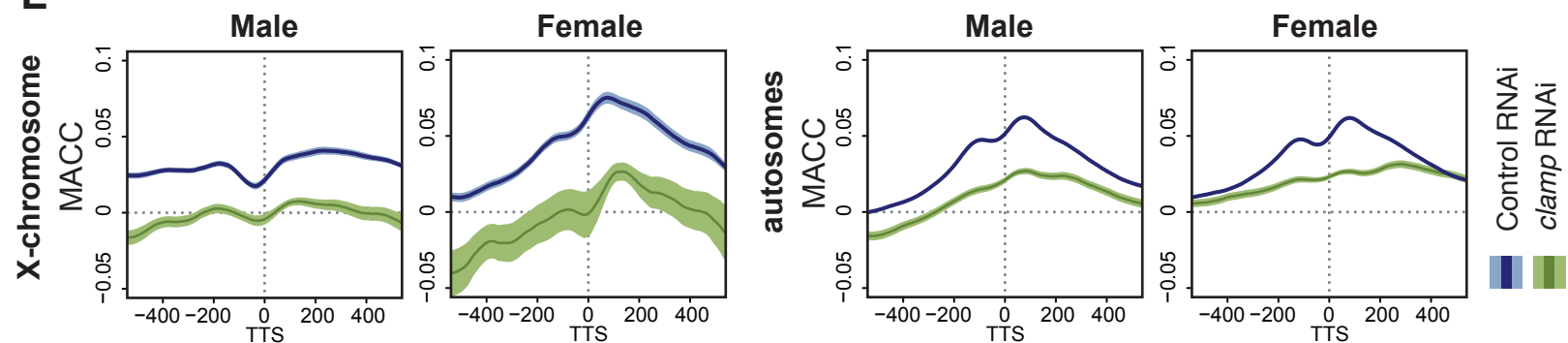**F**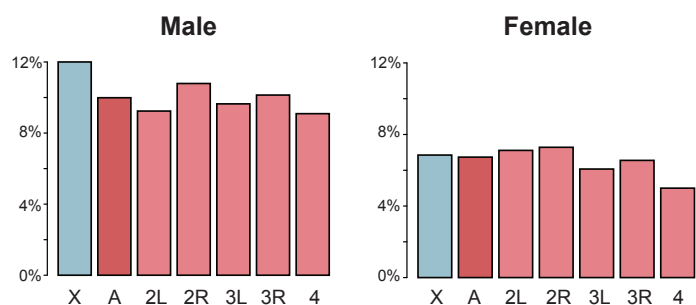**G**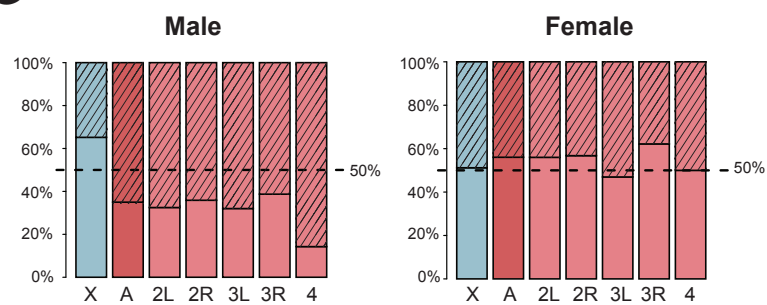

Supplement: S5 Fig — A) Average MACC profiles for genes with low enrichment of CLAMP are shown centered on transcription start sites (TSS) and separated into X-chromosome and autosome plots for male and female cells. RNAi treatment of clamp (green) and control (blue) is indicated, where the dark line represents the average MACC value, while 95% confidence intervals are represented by the lighter colors. B) The distribution of average MACC values around randomized TSS in non-repetitive regions is plotted for male (S2) cells. The darker line represents the average MACC value and the lighter shading indicates the 95% confidence interval. C and D) The nucleosome read counts obtained for each concentration of MNase are shown under control (blue) and clamp (green) RNAi conditions and centered over annotated TSS. Each concentration is shown as a gradient color of the RNAi treatment. Nucleosome profiles are shown for the male X-chromosome and autosomes separately for both males (C) and females (D). E) Average MACC values (darker line) were plotted +/- 500bp centered on transcription termination sites (TTS) separated by the X-chromosome and autosomes in males and females. There is a reduction in accessibility after clamp RNAi (green) compared to the control (blue). The lighter shading surrounding the mean line on all plots represents the 95% confidence interval. F) The percentages of significantly changed transcripts (p<0.05) in males (left) and females (right) are shown for each chromosomal arm. The blue bar indicates the X-chromosome and autosomes are in red/pink. G) The percentages of significantly changed transcripts (p<0.05) that decrease in abundance after clamp RNAi (un-hatched) or increase in abundance after clamp RNAi (hatched) are shown for each chromosomal arm in males (left) and females (right). (PDF) [file pone.0186855.s005.pdf]

**A****Nucleosome profiles in Males**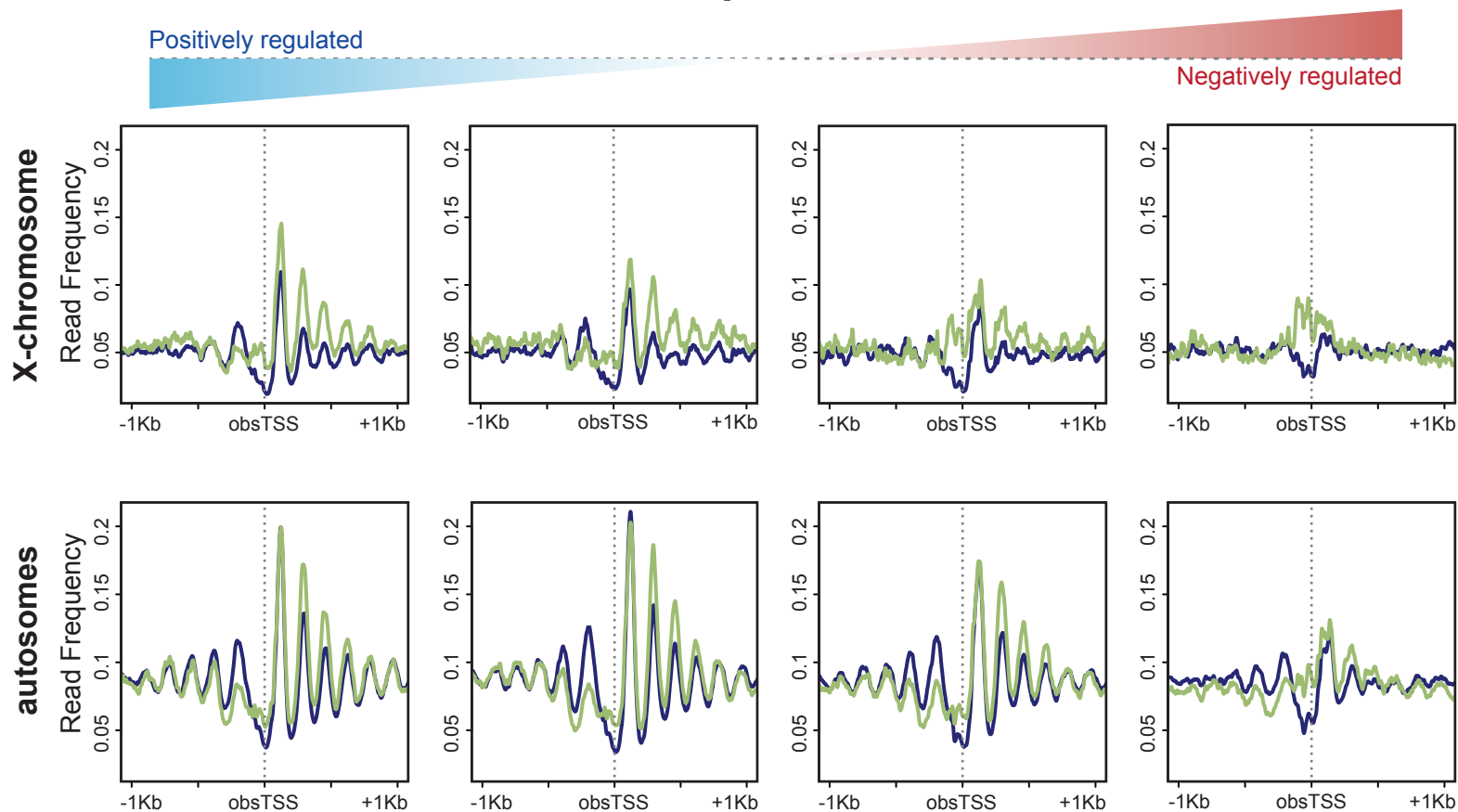**B****Nucleosome profiles in Females**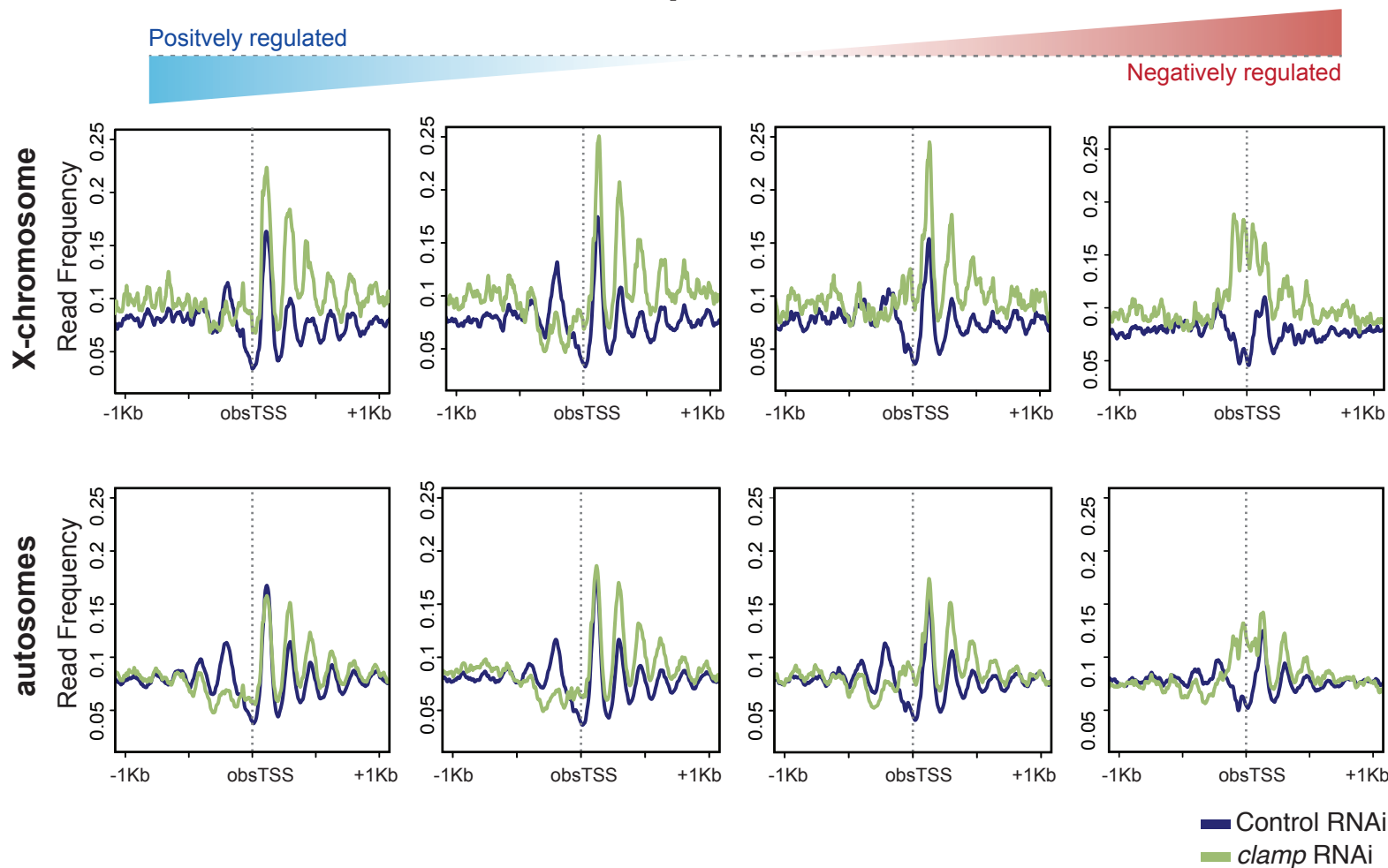

Supplement: S6 Fig — Profiles were generated for both males (A) and females (B). X-chromosome and autosome obsTSS were categorized into quartiles based on the ability of CLAMP to positively or negatively regulate transcription as measured by Start-seq. Shown are the profiles for each quartile. (PDF) [file pone.0186855.s006.pdf]

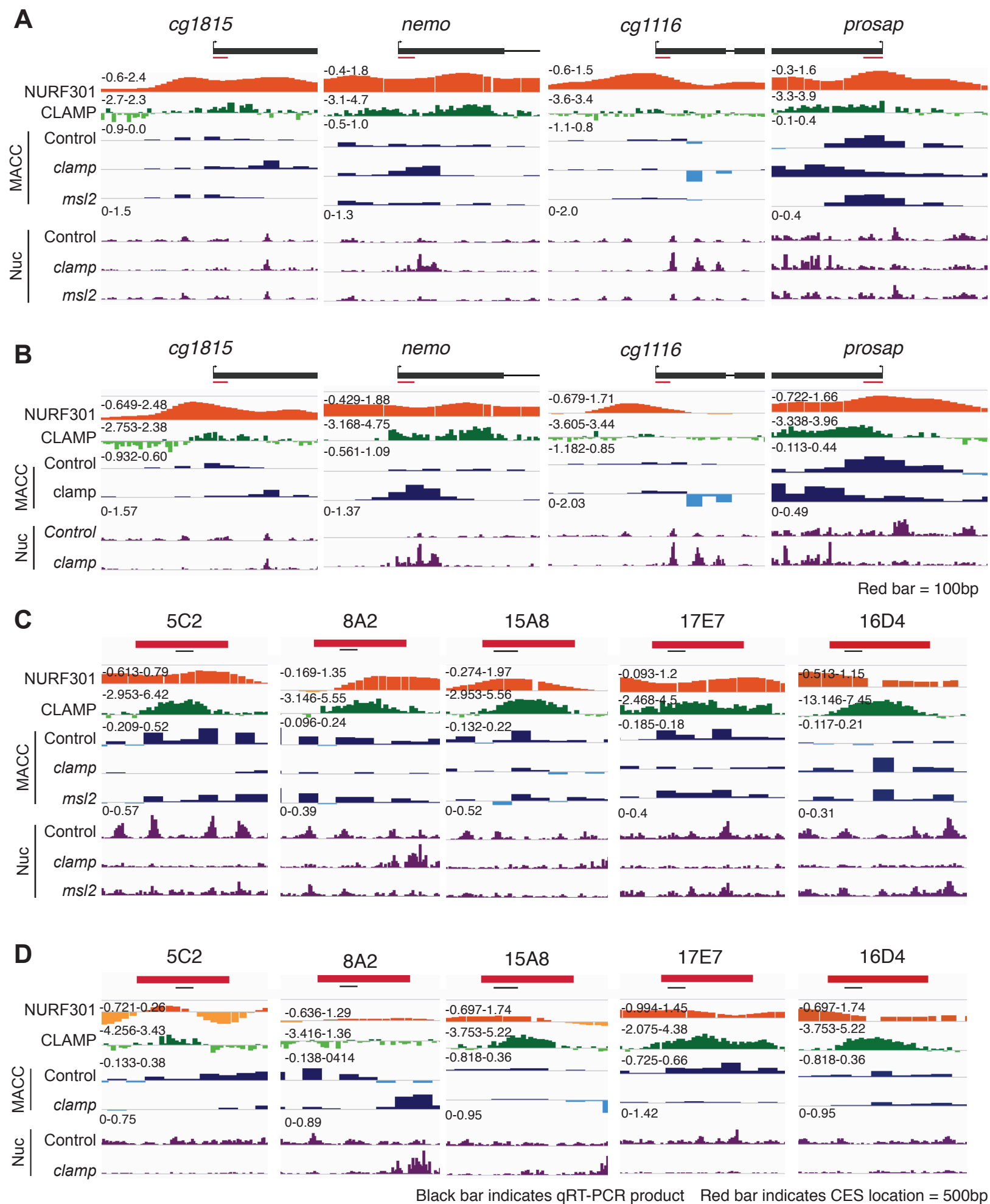

Supplement: S9 Fig — For each, enrichment for NURF301 (orange) and CLAMP (green) is shown. The MACC values after control, clamp, and msl2 RNAi treatment are shown in blue where dark blue indicates positive values and light blue are negative. The average number of sequencing reads from the four MNase-seq experiments generated a nucleosome profile that is shown in purple for Control, clamp, and msl2 RNAi. NURF301 recruitment was tested following clamp RNAi treatment by ChIP qRT-PCR at four promoters in males (A) and females (B), where the red bar underneath the gene is scaled to 100 bp. Similarly five CES were tested in males (C) and females (D). The black bar underneath the red CES bar indicates the location of the qRT-PCR product for the ChIP qPCR experiments. (PDF) [file pone.0186855.s009.pdf]
