# Supplementary material for: Enhanced chromatin accessibility of the dosage compensated Drosophila male X-chromosome requires the CLAMP zinc finger protein
Source: PLoS One. 2017 Oct 27;12(10):e0186855. doi: 10.1371/journal.pone.0186855 (PMC5659772; doi:10.1371/journal.pone.0186855)

Nucleosome profiles in Females

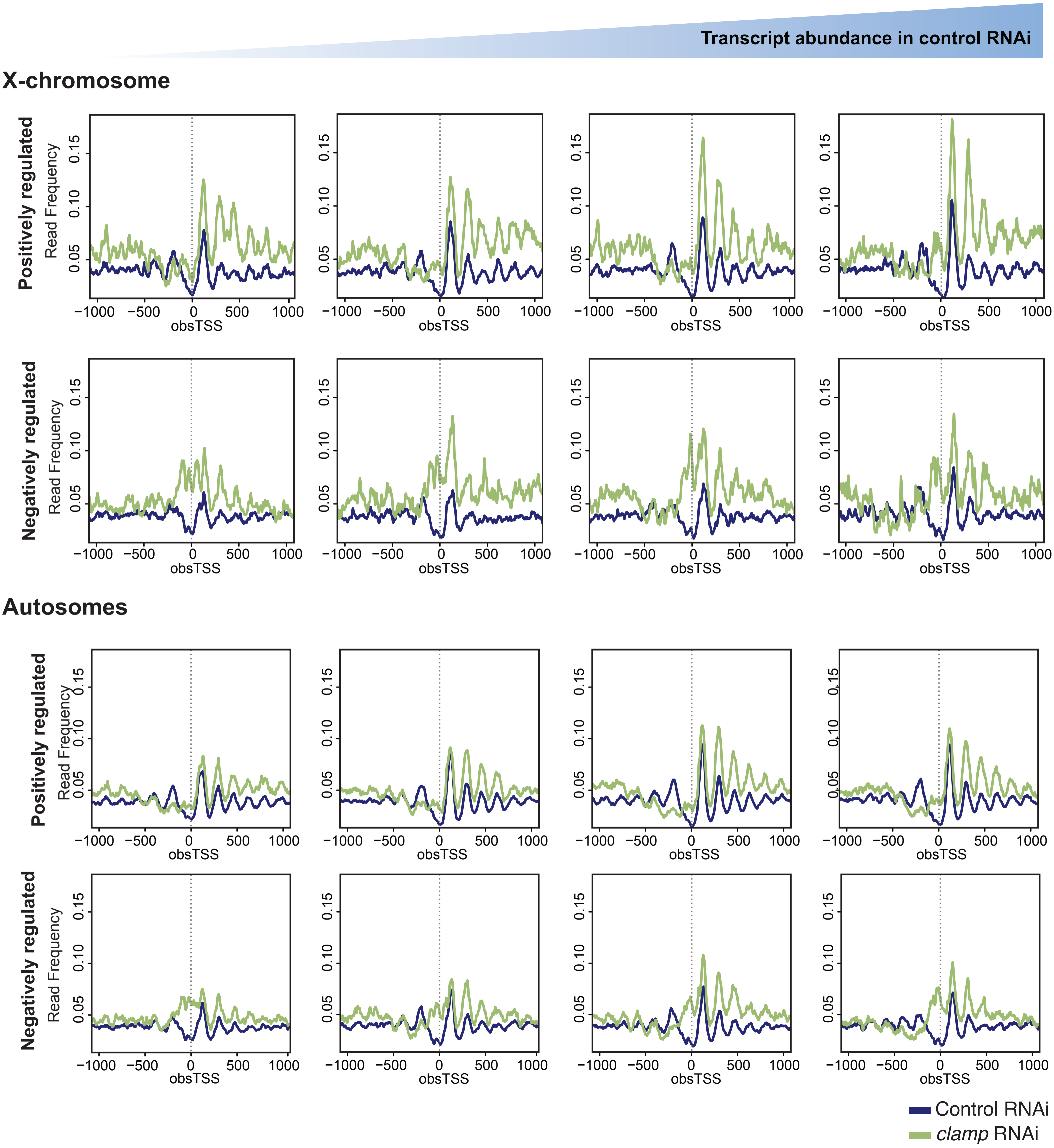

Supplement: S8 Fig — X-chromosome and autosome obsTSS were categorized into quartiles of increasing expression level as determined by transcript abundance in the control RNAi condition and separated by whether they are positively or negatively regulated by CLAMP. (PDF) [file pone.0186855.s008.pdf]
