# Supplementary material for: Enhanced chromatin accessibility of the dosage compensated Drosophila male X-chromosome requires the CLAMP zinc finger protein
Source: PLoS One. 2017 Oct 27;12(10):e0186855. doi: 10.1371/journal.pone.0186855 (PMC5659772; doi:10.1371/journal.pone.0186855)
Supplement: S1 Table — (PDF) [file pone.0186855.s010.pdf]

S1 Table (Urban,Kuzu)

A Mann-Whitney-Wilcoxon test p-values for Figure 1B

	condition 1	condition 2	p-value
**Males**	control X	control A	< 2.2e-16
		msl2 X	0.001529
		msl2 A	< 2.2e-16
		clamp X	< 2.2e-16
		clamp A	< 2.2e-16
	control A	*msl2* X	< 2.2e-16
		msl2 A	1.16E-10
		clamp X	< 2.2e-16
		clamp A	0.9339
	msl2 X	*msl2* A	< 2.2e-16
		clamp X	< 2.2e-16
		clamp A	< 2.2e-16
	msl2 A	*clamp* X	< 2.2e-16
		clamp A	2.20E-07
	clamp X	*clamp* A	< 2.2e-16
**Females**	control X	control A	< 2.2e-16
		clamp X	< 2.2e-16
		clamp A	< 2.2e-16
	control A	*clamp* X	0.1895
		clamp A	< 2.2e-16
	clamp X	*clamp* A	< 2.2e-16

B Mann-Whitney-Wilcoxon test p-values for Figure 1C

condition 1	condition 2	p-value
S2 *msl2* X	S2 *msl2* A	< 2.2e-16
	S2 *clamp* X	< 2.2e-16
	S2 *clamp* A	< 2.2e-16
	Kc *clamp* X	< 2.2e-16
	Kc *clamp* A	< 2.2e-16
S2 *msl2* A	S2 *clamp* X	< 2.2e-16
	S2 *clamp* A	< 2.2e-16
	Kc *clamp* X	< 2.2e-16
	Kc *clamp* A	< 2.2e-16
S2 *clamp* X	S2 *clamp* A	< 2.2e-16
	Kc *clamp* X	< 2.2e-16
	Kc *clamp* A	< 2.2e-16
S2 *clamp* A	Kc *clamp* X	0.0122
	Kc *clamp* A	< 2.2e-16
Kc *clamp* X	Kc *clamp* A	< 2.2e-16

C Mann-Whitney-Wilcoxon test p-values Figure 2 All CES

condition 1	condition 2	p-value
S2 control	S2 *clamp*	< 2.2e-16
	S2 *msl2*	7.41E-11
	Kc control	0.388
	Kc *clamp*	< 2.2e-16
S2 *clamp*	S2 *msl2*	< 2.2e-16
	Kc control	< 2.2e-16
	Kc *clamp*	< 2.2e-16
S2 *msl2*	Kc control	7.89E-14
	Kc *clamp*	< 2.2e-16
Kc control	Kc *clamp*	< 2.2e-16

D Mann-Whitney-Wilcoxon test p-values Figure 3C

	condition 1	condition 2	p-value
**X versus A**	S2 *msl2* X	S2 *msl2* A	< 2.2e-16
		S2 *clamp* X	< 2.2e-16
		S2 *clamp* A	1.14E-07
		Kc *clamp* X	0.001638
		Kc *clamp* A	1.78E-12
	S2 *msl2* A	S2 *clamp* X	< 2.2e-16
		S2 *clamp* A	0.000393
		Kc *clamp* X	1.66E-10
		Kc *clamp* A	3.90E-15
	S2 *clamp* X	S2 *clamp* A	< 2.2e-16
		Kc *clamp* X	< 2.2e-16
		Kc *clamp* A	< 2.2e-16
	S2 *clamp* A	Kc *clamp* X	3.75E-12
		Kc *clamp* A	0.000261
	Kc *clamp* X	Kc *clamp* A	3.42E-16
**All**	S2-*msl2*	S2 *clamp*	< 2.2e-16
		Kc *clamp*	1.37E-09
	S2-*clamp*	Kc *clamp*	< 2.2e-16

E Mann-Whitney-Wilcoxon test p-values Figure 2 and S3

condition 1	condition 2	Group A	Group B	Group C	Random (S3C)	pionX (S3D)
S2 control	S2 *clamp*	< 2.2e-16	9.10E-13	1.50E-04	3.10E-02	1.60E-06
	S2 *msl2*	1.10E-13	1.00E-05	8.00E-02	4.40E-01	7.10E-02
S2 *clamp*	S2 *msl2*	< 2.2e-16	6.80E-05	1.10E-03	1.30E-01	5.50E-04
Kc control	Kc *clamp*	9.50E-07	1.70E-03	7.00E-01		
