# Supplementary material for: Enhanced chromatin accessibility of the dosage compensated Drosophila male X-chromosome requires the CLAMP zinc finger protein
Source: PLoS One. 2017 Oct 27;12(10):e0186855. doi: 10.1371/journal.pone.0186855 (PMC5659772; doi:10.1371/journal.pone.0186855)
Supplement: S2 Table — (PDF) [file pone.0186855.s011.pdf]

S2 Table (Urban,Kuzu)

A

P-values for H3 ChIP		
	Male	Female
CES5C2	0.029999	0.523343
CES15A8	0.355948	0.175951
CES8A2	0.098759	0.508723
CES17E7	0.668788	0.070262
CES16D4	0.539125	0.11042
cg1116	0.401524	0.788447
cg1815	0.528867	0.221609
Nemo	0.560269	0.098956
Prosap	0.415684	0.079605

B

P-values for NURF ChIP		
	Male	Female
CES5C2	0.131823	0.276855
CES15A8	0.210989	0.071625
CES8A2	0.616248	0.519939
CES17E7	0.432081	0.385921
CES16D4	0.819964	0.212601
cg1116	0.813449	0.535593
cg1815	0.26526	0.028105
Nemo	0.849661	0.84194
Prosap	0.493223	0.604134
