# Supplementary material for: Enhanced chromatin accessibility of the dosage compensated Drosophila male X-chromosome requires the CLAMP zinc finger protein
Source: PLoS One. 2017 Oct 27;12(10):e0186855. doi: 10.1371/journal.pone.0186855 (PMC5659772; doi:10.1371/journal.pone.0186855)
Supplement: S3 Table — (PDF) [file pone.0186855.s012.pdf]

S3 Table (Urban,Kuzu)

		Forward	Reverse
ChIP	*cg1815*	TCT TTG TCT GCT CAT GAA TTT CGA	TGG TCG AGG GAA ACT TTG CT
	nemo	GTG CGG GCS CTC TAA AAA CAT	GGT CGG CCA CCG ATA GAA AC
	cg1116	AAG GCC TAC CTG GGA TTC TG	GCG TAC GCC AAC CAC TTT TT
	prosap	TAC AGG CCG ATA ACG TTG GG	AAG TGC TGC CTC AAA CCT CA
	CES5C2	ATC AAT GTT TCG ATG TAG A	CTT CCA ACT ATC TCG CTC T
	CES8A2	TAA TGT CCG CGA GAA ATG TG	AAG TGC AGA AGG CAT TGG TT
	CES15A8	TGACGCCTTTGCTGAATGT	TGCGCTCCTATCACCCAGA
	CES17E7	TAT TTT CAA CAT GCG CGA AG	ATT CAC TCT TGG CCG ACA AC
	CES16D4	AGC CTA GCC CCC ATA TAG TTG AGG T	GAC CTT TGG GCA CGG GCG TT
dsRNA	*gfp*	TAA TAC GAC TCA CTA TAG GGG GTG AGC AAG GGC GAG GAG CT	TAA TAC GAC TCA CTA TAG GGT CTT GAA GTT CAC CTT GAT GCC G
	clamp	TAA TAC GAC TCA CTA TAG GGG GGC GGC GAT TCT ATT G	TAA TAC GAC TCA CTA TAG GGC ATG GGA GTG CTG CCC C
	msl2	TAA TAC GAC TCA CTA TAG GGT GTT GGC TCG TCA CTG TCC TC	TAA TAC GAC TCA CTA TAG GGG TTG GCT GTG CTG GCT GCC GT
RNA	*clamp*	CTG ACC ACG GCA ATA GAA	TCC CTC ATT TGG CAC TTC
	msl2	GCC CTG TCC GTA TGA ATG	CAA CAT GGG TAA ACA ACC
	roX2	CGT TAC TCT TGC TTG ATT TTG C	AGCTCGGATGGCCATCGAAA
